# Supplementary material for: Conformational dynamics of cohesin/Scc2 loading complex are regulated by Smc3 acetylation and ATP binding
Source: Nat Commun. 2023 Sep 22;14:5929. doi: 10.1038/s41467-023-41596-w (PMC10516938; doi:10.1038/s41467-023-41596-w)
Supplement: Supplementary file 1 — Supplementary Information [file 41467_2023_41596_MOESM1_ESM.pdf]

Supplementary Information

Conformational dynamics of cohesin/Scc2 loading complex are regulated by Smc3 acetylation and ATP binding

Aditi Kaushik, Thane Than, Naomi J Petel3, Menelaos Voulgari3, Charlotte Percival, Peter Daniels, John B Rafferty, Kim A Nasmyth, and Bin Hu

Inventory of Supplementary Information

Supplementary Fig 1. smc3-R1008I barely suppress the lethality of smc3-QQ

Supplementary Fig 2. *smc3-QQ* alters neither cohesin's interaction with DNA nor Smc3-Smc1's head engagement.

Supplementary Fig 3. Scc2 interacts with both Smc3 and Smc1's heads

Supplementary Fig 4. Smc3 Q67/Scc2 T1175 in a unique interface

Supplementary Fig 5. ATP hydrolysis mutant does not affect Smc3 Q67/Scc2 T1175 interaction

Supplementary Fig 6. Inhibition in Smc3 Q67/Scc2 T1175 interaction by *smc3-QQ* is independent of Pds5

Supplementary Fig 7. *smc3-R1008I / R1008I_W483R* recovers crosslink efficiencies between Smc3/Scc2 interfaces

Supplementary Table 1 Cysteine pairs used in this study

Supplementary Table 2 Mutations used in this study

Supplementary Table 3 yeast strains used in this study

Supplementary Table 4 Positions of yeast CDE III

Supplementary Table 5 Antibodies and Reagents

Supplementary Figure 1

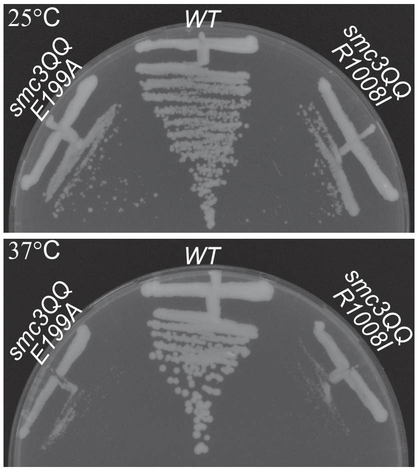

Supplementary Fig 1 *smc3-R1008I* barely suppress the lethality of *smc3-QQ*. The growth of indicated strains after incubation on YPD plates at 25°C or 37°C for two days. Source data is provided as a Source Data file.

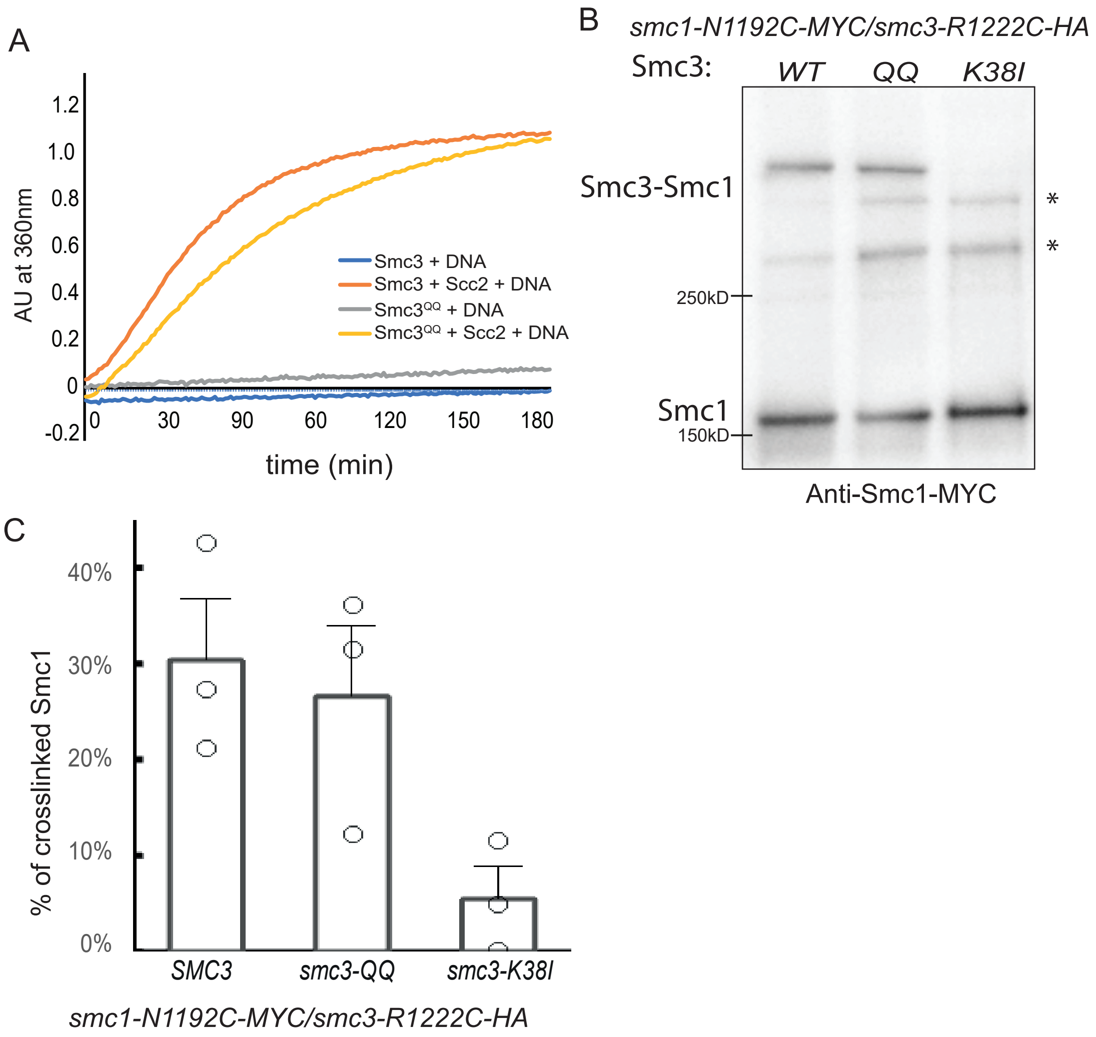

Supplementary Fig 2 *smc3-QQ* alters neither cohesin's interaction with DNA nor Smc3-Smc1's head engagement
(A) Recombinant tetramer of cohesin (Smc1, Smc3 or Smc3^{QQ}, Scc1 and Scc3) was incubated with DNA or Scc2 or both. ATP was added to initiate the reaction, and the reaction rate was measured as the change in absorption at 360nm over time. Source data is provided as a Source Data file.
(B) *In vivo* BMOE crosslink between *smc1 N1192C* and *smc3 R1222C* representing the head engagement between Smc1 and Smc3. Cohesin complexes were immunoprecipitated using anti-PK antibody for Scc1-PK from the exponentially grown *SMC3*, *smc3-QQ* or *smc3-K38I* cells. Crosslink bands were separated by 3-8% gradient gel and non-crosslinked and crosslinked Smc1 was examined by Western Blot using anti-Myc antibody. Asterisks show the positions of non-specific bands. Source data is provided as a Source Data file.
(C) The percentage of crosslinking efficiency between Smc1 and Smc3 heads was calculated as mean+SD from 3 independent experiments based on anti-MYC western blot, as shown on panel B. Source data is provided as a Source Data file.

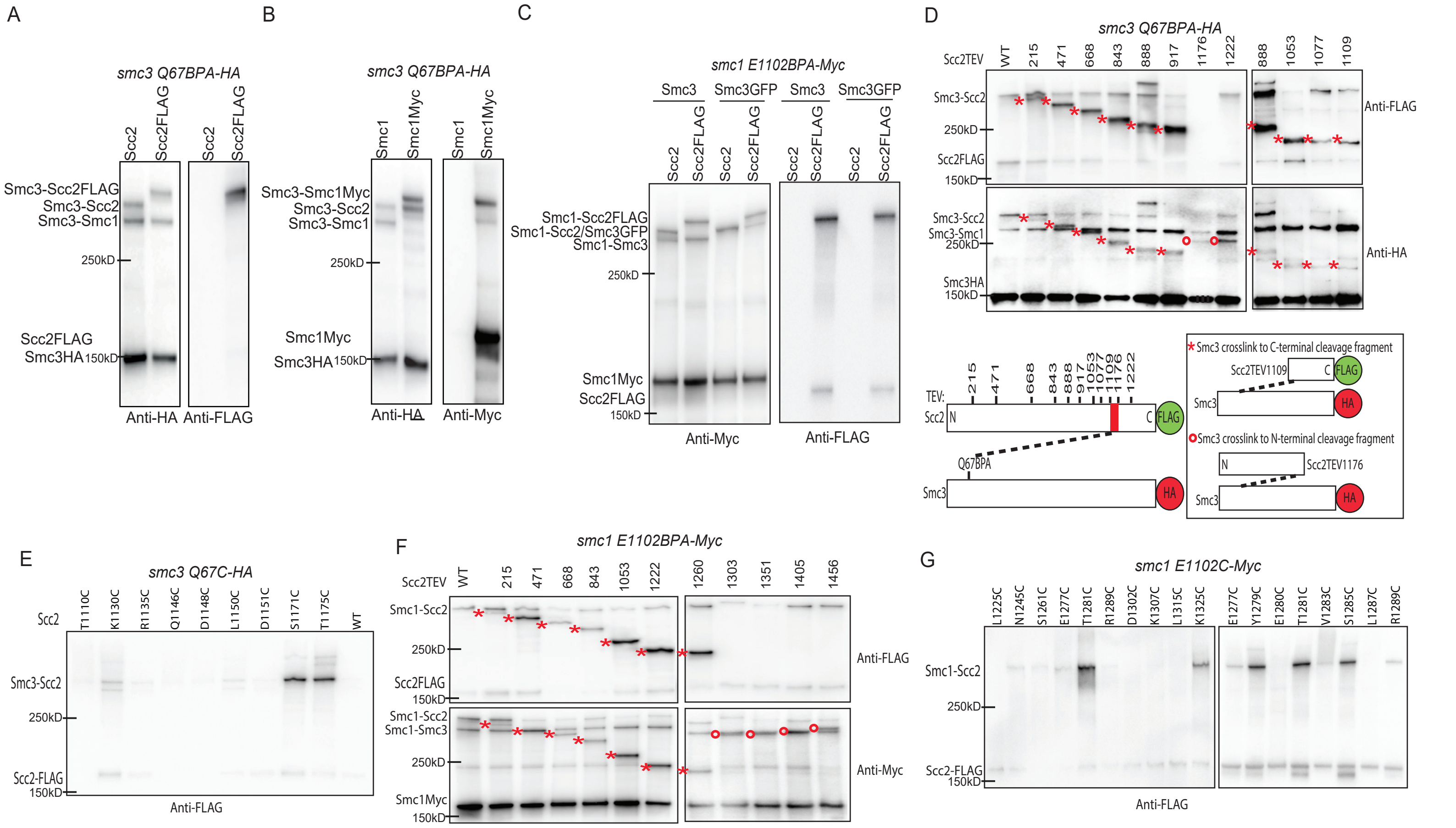

Supplementary Fig 3 Scc2 interacts with both Smc3 and Smc1's heads

(A) *In vivo* BPA crosslink of *smc3 Q67BPA* in the exponentially grown cells. Scc1-PK was immunoprecipitated from whole-cell extracts and co-immunoprecipitated Smc3Q67BPA-HA, and Scc2-FLAG was detected in western Blot. Fusing Scc2 with 6xFLAG confirmed the top band as an Smc3-Scc2 crosslink product. Source data is provided as a Source Data file.

(B) *In vivo* BPA crosslink of *smc3 Q67BPA* in the exponentially grown cells. Scc1-PK was immunoprecipitated from whole-cell extracts and co-immunoprecipitated Smc3^{Q67BPA}-HA, and Smc1-MYC were detected by western blot using anti-MYC antibody. Fusing Smc1 with 9xMyc confirmed the second band as an Smc3-Smc1 crosslink product. Source data is provided as a Source Data file.

(C) *In vivo* BPA crosslink of *smc1 E1102BPA* in the exponentially grown cells. Scc1-PK was immunoprecipitated from whole-cell extracts and co-immunoprecipitated Smc1^{E1102BPA}-MYC and Scc2-FLAG were detected by Western blot using anti-MYC and anti-FLAG antibodies. Fusing Smc3 with GFP and Scc2 with 6xFLAG revealed the top crosslink band to be Smc1-Scc2 and the lower to be Smc1-Smc3. Source data is provided as a Source Data file.

(D) The Smc3^{Q67BPA}-HA/Scc2^{TEV}-FLAG crosslink proteins were co-immunoprecipitated with Scc1-PK from the exponentially grown cells. Then the crosslink proteins were digested by TEV protease, and cleaved fragments were examined by Western Blot using anti-HA and anti-FLAG antibody. All the samples derive from the same experiment and both Western blots were processed in parallel. Source data is provided as a Source Data file.

(E) The BMOE crosslink of *smc3 Q67C* with indicated cysteine substituted Scc2. Scc1-PK was immunoprecipitated from the exponentially grown cells and co-immunoprecipitated Scc2 was detected using an anti-FLAG antibody. Source data is provided as a Source Data file.

(F) The Smc1^{E1102BPA}-MYC/Scc2^{TEV}-FLAG crosslink proteins were co-immunoprecipitated with Scc1-PK from the exponentially grown cells. Then the crosslink proteins were digested by TEV protease, and cleaved fragments were examined by Western Blot using anti-MYC and anti-FLAG antibody. All the samples derive from the same experiment and both Western blots were processed in parallel. Source data is provided as a Source Data file.

(G) The BMOE crosslink of *smc1 E1102C* with indicated cysteine substituted Scc2 in the exponentially grown cells. Scc1-PK was immunoprecipitated from whole-cell extracts and co-immunoprecipitated Scc2 was detected using anti-FLAG antibody. All the samples derive from the same experiment and both Western blots were processed in parallel. Source data is provided as a Source Data file.

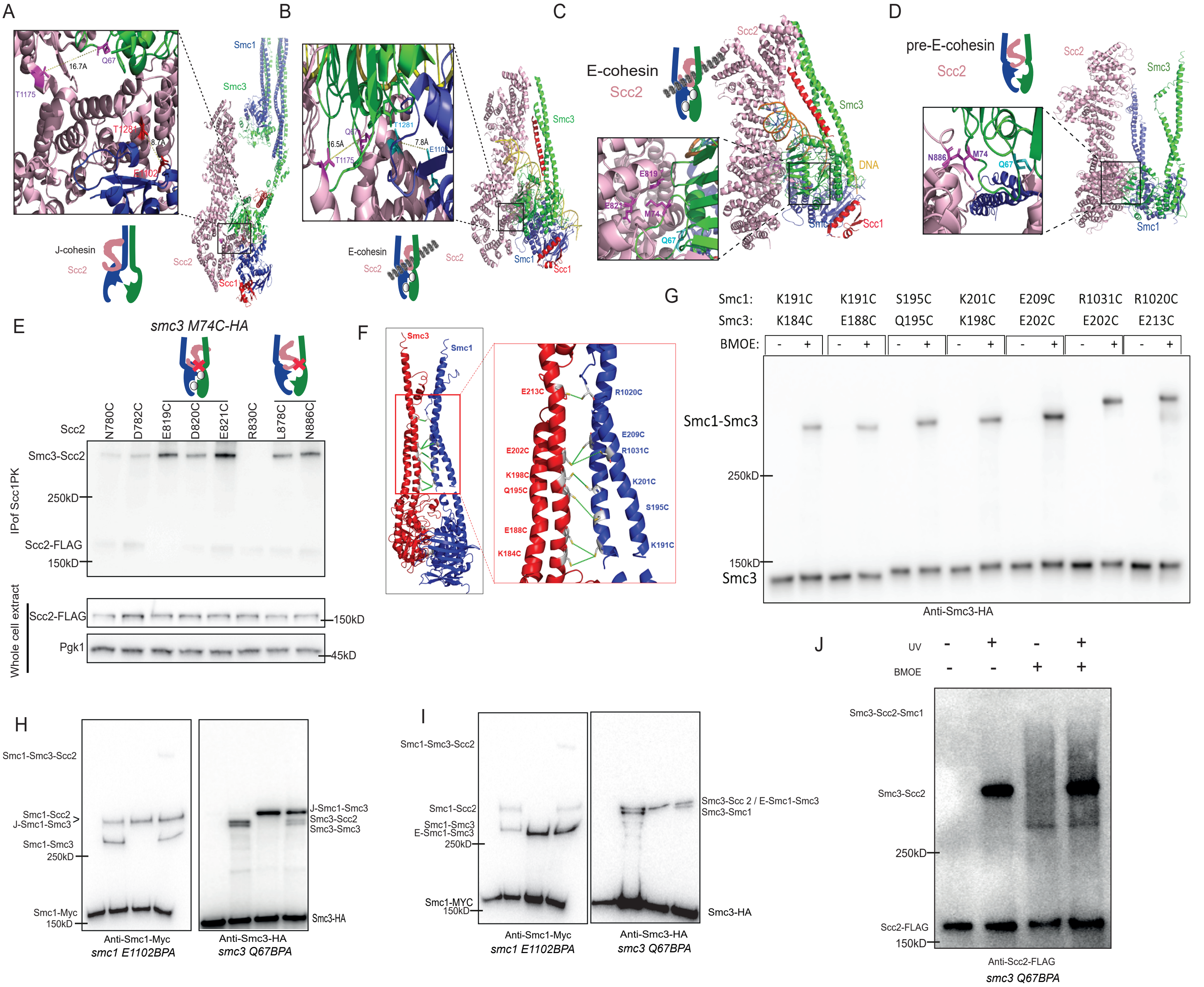

Supplementary Fig 4 Smc3 Q67/Scc2 T1175 in a unique interface

(A) The distances between Smc3 Q67/Scc2 T1175 or Smc1 E1102/Scc2T1281 were indicated in the cryo-EM structure of the Scc2/J-cohesin complex (EMD-12880 [https://pdbj.org/emnavi/quick.php?id=emdb-12880]).

(B) The distances between Smc3 Q67-Scc2 T1175C or Smc1 E1102- Scc2T1281 were indicated in the cryo-EM structure of the Scc2/E-cohesin complex (PDB 6ZZ6 [https://www.rcsb.org/structure/6zz6]).

(C) The relative positions of Smc3 M74 and Smc3 Q67 with Scc2 E819 and Scc2 E821 were indicated in the cryo-EM structure of the Scc2/E-cohesin complex (PDB 6ZZ6 [https://www.rcsb.org/structure/6zz6]).

(D) The relative positions of Smc3 M74 and Smc3 Q67 with Scc2 N888 in the Scc2/pre-E-cohesin complex.

(E) The *in vivo* BMOE crosslink of *smc3 M74C* with indicated cysteine substituted Scc2. Scc1-PK was immunoprecipitated from exponentially grown cells and co-immunoprecipitated Scc2-FLAG was detected by Western Blot using anti-FLAG Antibody. Whole-cell extract showing equal expressions of Scc2-FLAG for all the strains with the loading control PGK1. Source data is provided as a Source Data file.

(F) Structural model of juxtaposed cohesin coiled-coils and the positions of residues used for crosslink to stabilise J-cohesin.

(G) *In vivo* BMOE crosslink of indicated cysteine pairs on cohesin coiled-coils. Scc1 was immunoprecipitated from exponentially grown cells using Anti-PK antibody, and co-immunoprecipitated Smc3 (non-crosslinked and crosslinked to Smc1) was analysed using an anti-HA antibody. Source data is provided as a Source Data file.

(H) *In vivo* BPA crosslink of *smc1 E1102BPA* (left) or *smc3 Q67BPA* (right) to Scc2 in J-cohesin using exponentially grown cells. Juxtaposed coiled-coils were stabilised by *in vivo* BMOE crosslink between *smc1 R1031C* and *smc3 E202C*, followed by UV-induced BPA crosslink of *smc1 E1102BPA* or *smc3 Q67BPA* with Scc2. Scc1-PK was immunoprecipitated from whole-cell extracts, and co-immunoprecipitated Smc1 (left) or Smc3 (right) was detected by anti-Myc or anti-HA antibody. Source data is provided as a Source Data file.

(I) *In vivo* BPA crosslink of *smc1 E1102BPA* (left) or *smc3 Q67BPA* (right) to Scc2 in E-cohesin using exponentially grown cycling cells. Engaged heads were stabilised by *in vivo* BMOE crosslink between *smc1 N1192C* and *smc3 R1222C*, followed by UV-induced BPA crosslink of *smc1 E1102BPA* or *smc3 Q67BPA* with Scc2. Scc1-PK was immunoprecipitated from whole-cell extracts and co-immunoprecipitated Smc1 or Smc3 was detected by anti-Myc or anti-HA antibody. Source data is provided as a Source Data file.

(J) Overexposed blot for Fig 4D, right hand side (*smc3 Q67BPA*/Scc2). Source data is provided as a Source Data file.

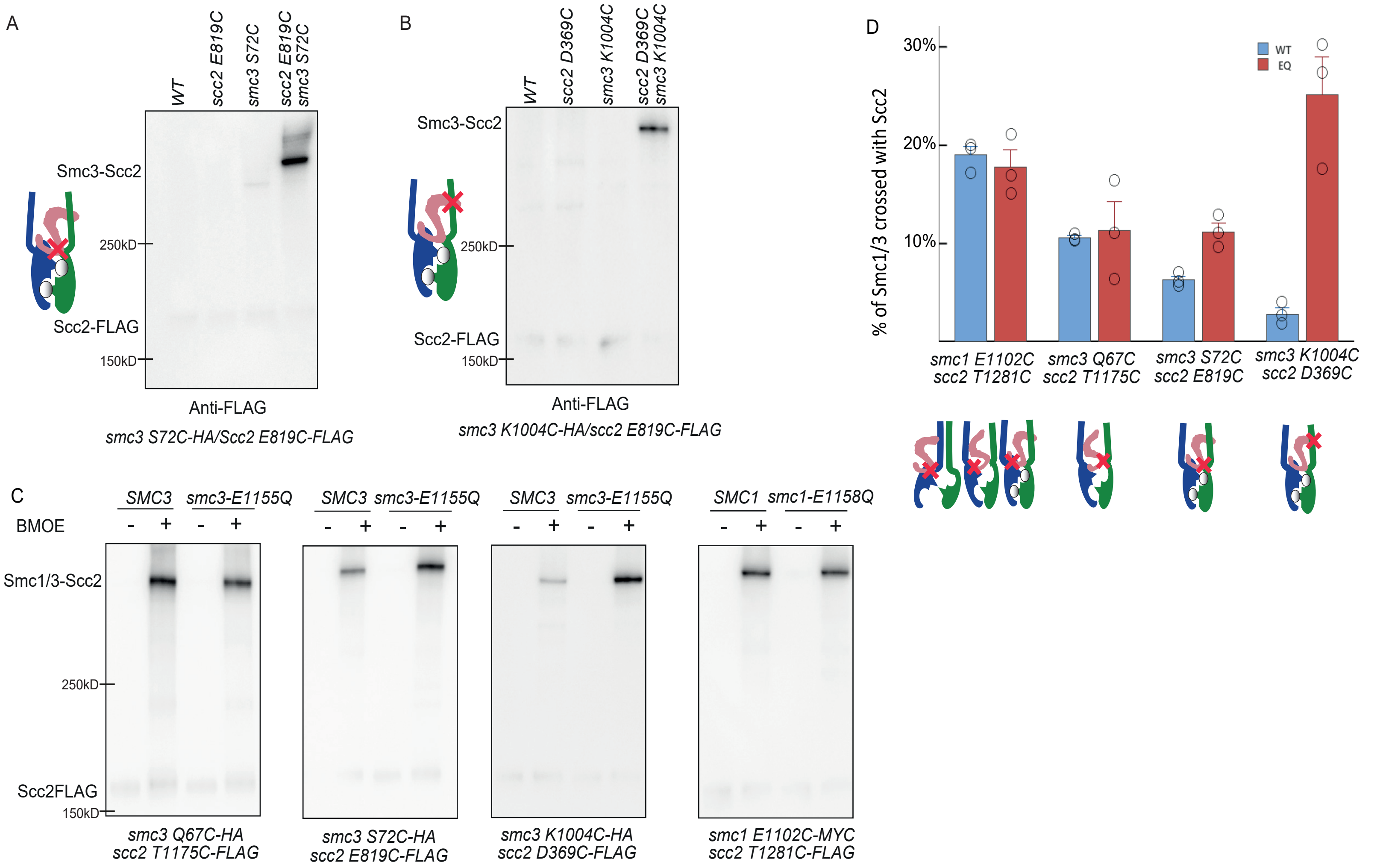

Supplementary Fig 5 ATP hydrolysis mutant does not affect Smc3 Q67/Scc2 T1175 interaction

(A) *In vivo* BMOE crosslinking of *smc3 S72C/scc2 E819C* in exponentially grown cycling cells. Scc1-PK was immunoprecipitated from whole-cell extracts, co-immunoprecipitated Scc2-FLAG was detected by Western blot using an anti-FLAG antibody. Successful crosslink product formation depended on the presence of both cysteines. Source data is provided as a Source Data file.

(B) *In vivo* BMOE crosslinking between *smc3 K1004C/scc2 D369C* in exponentially grown cycling cells. Scc1-PK was immunoprecipitated from whole-cell extracts, co-immunoprecipitated Scc2-FLAG was detected by western blot using an anti-FLAG antibody. Successful crosslink product formation depended on the presence of both cysteines. Source data is provided as a Source Data file.

(C) Effect of cohesin ATP hydrolysis mutant (*smc3-E1155Q* or *smc1-E1158Q*) on the *in vivo* BMOE crosslinks on different Scc2/cohesin interfaces. Ectopically expressed wild type or EQ mutant versions of *smc3 S72C*, *smc3 K1004C, smc3 Q67C*, or *smc1 E1102C* were crosslinked to their respective cysteine substitution of Scc2 residues using BMOE in late G1 arrested cells. Scc1-PK was immunoprecipitated from whole-cell extracts and coimmunoprecipitated Scc2 was detected by western blot using anti-FLAG antibody. Crosslinking efficiency was quantified using band intensity on western blot. Source data is provided as a Source Data file.

(D) The percentage of crosslinking efficiency in Fig 5D was calculated as mean+SD from 3 independent experiments. *smc3-E1155Q* increased the crosslinking efficiency of Scc2 with *smc3 S72C* and *smc3 K1004C*. The highest increase of about 10-fold was seen in the crosslink *smc3 K1004C/scc2 D369C* crosslink. Source data is provided as a Source Data file.

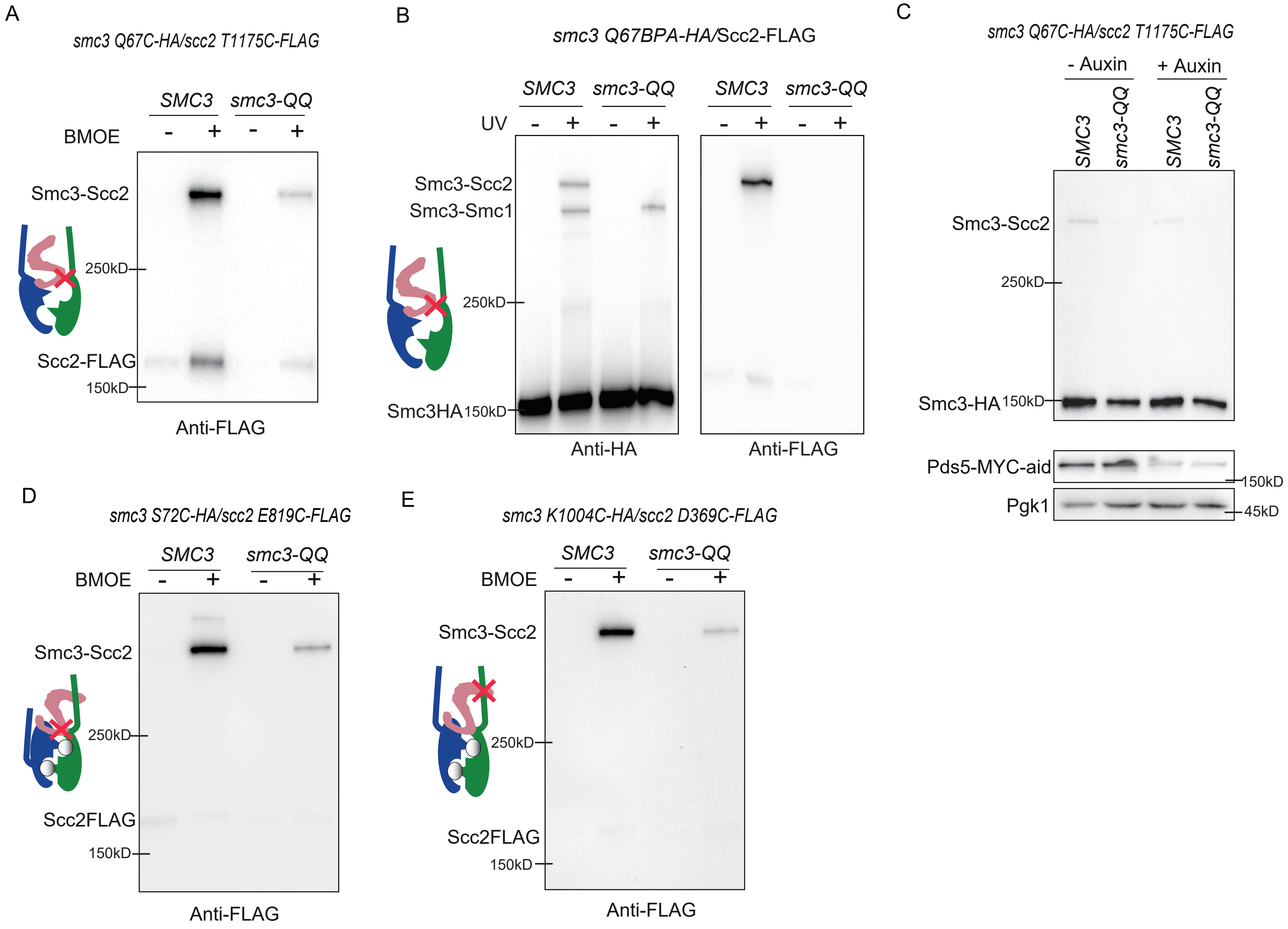

Supplementary Fig 6 Inhibition in Smc3 Q67/Scc2 T1175 interaction by smc3-QQ is independent of Pds5

(A) *In vivo* BMOE crosslink of *scc2 T1175C* with *smc3* or *smc3-QQ Q67C* using cells arrested in late G1. Scc1-PK was immunoprecipitated from whole-cell extracts, and co-immunoprecipitated Scc2-FLAG was detected by western blot using anti-FLAG antibody. Source data is provided as a Source Data file.

(B) *In vivo* BPA crosslink of Scc2 with *smc3* or *smc3-QQ Q67BPA* in exponentially grown cycling cells. Scc1-PK was immunoprecipitated from whole-cell extracts, and co-immunoprecipitated Scc2-FLAG was detected by western blot using anti-FLAG antibody. Source data is provided as a Source Data file.

(C) *In vivo* BMOE crosslink between *smc3* or *smc3-QQ Q67C* /*scc2 T1175C* in the presence or absence of Auxin-included degradable Pds5. Cohesin complex was immunoprecipitated using anti-PK antibody from exponentially grown cells, and western blot was performed to analyse Smc3-HA. Whole-cell extract showing Auxin-induced degradation of Pds5-MYC9 and loading control PGK1. Source data is provided as a Source Data file.

(D) *In vivo* BMOE crosslink of *scc2 E819C* with *smc3* or *smc3-QQ S72C* using cells arrested in late G1. Scc1-PK was immunoprecipitated from whole-cell extracts, and co-immunoprecipitated Scc2-FLAG was detected by western blot using anti-FLAG antibody. Source data is provided as a Source Data file.

(E) *In vivo* BMOE crosslink of *scc2 D369C* with *smc3* or *smc3-QQ K1004C* using cells arrested in late G1. Scc1-PK was immunoprecipitated from whole-cell extracts, and co-immunoprecipitated Scc2-FLAG was detected by western blot using anti-FLAG antibody. Source data is provided as a Source Data file.

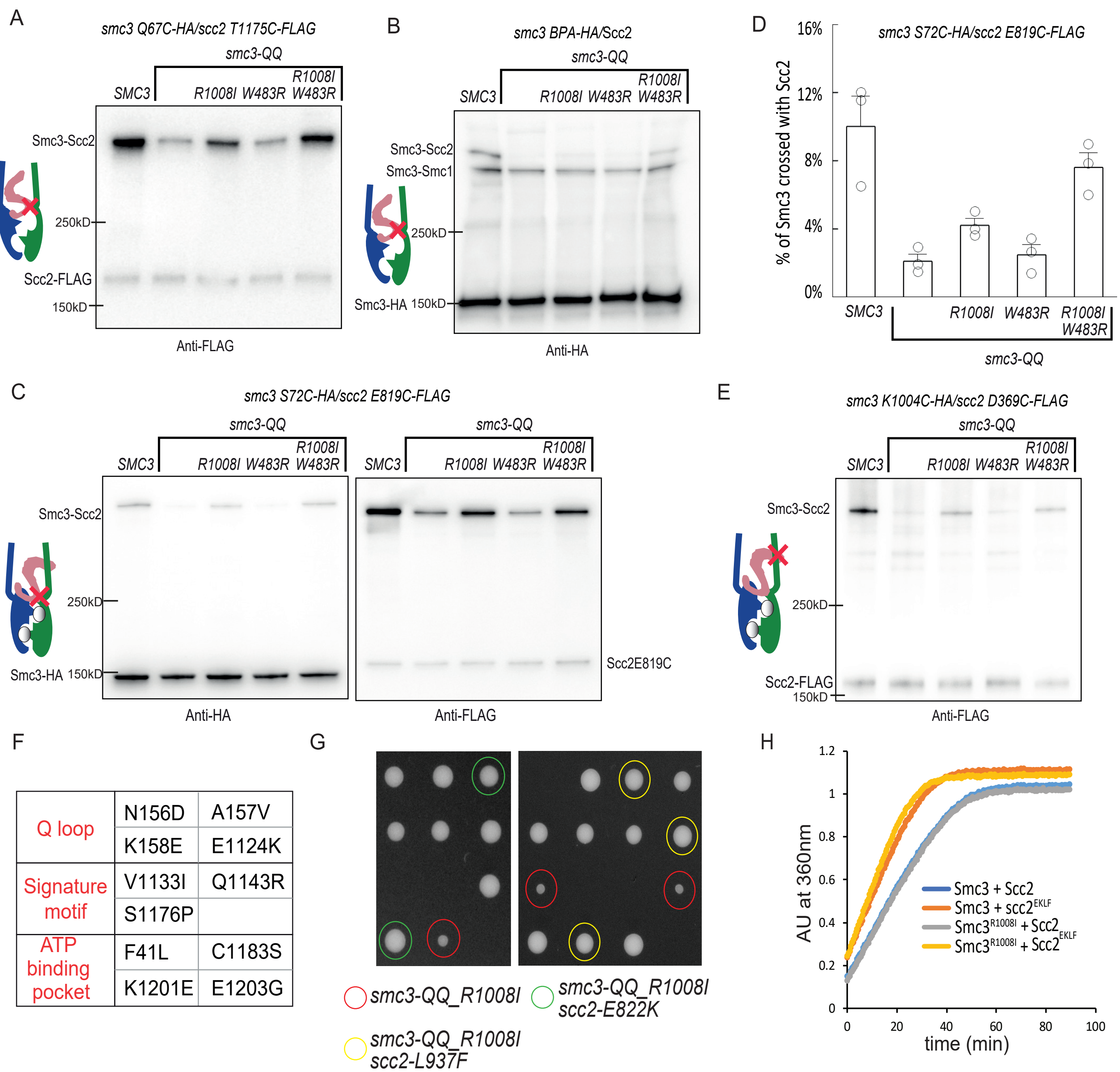

Supplementary Fig 7 *smc3-R1008I* / *R1008I_W483R* recovers crosslink efficiencies between Smc3/Scc2 interfaces

(A) *In vivo* BMOE crosslink between *scc2 T1175C* and *smc3-QQ Q67C* in the presence of *smc3 R1008I* or *W483R* or both using cells arrested in late G1. Scc1-PK was immunoprecipitated by anti-PK antibody and co-immunoprecipitated Scc2-FLAG was detected by western blot using an anti-FLAG antibody. Source data is provided as a Source Data file.

(B) *In vivo* BPA crosslink between Scc2 and *smc3-QQ Q67BPA* in the presence of *smc3 R1008I* or *W483R* or both in exponentially grown cells. Scc1-PK was immunoprecipitated from whole-cell extracts and co-immunoprecipitated Scc2-FLAG was detected by western blot using an anti-FLAG antibody. Source data is provided as a Source Data file.

(C) *In vivo* BMOE crosslink between *scc2 E819C* and *smc3-QQ S72C* in the presence of *smc3 R1008I* or *W483R* or both using cells arrested in late G1. Scc1-PK was immunoprecipitated and co-immunoprecipitated Smc3-HA and Scc2-FLAG was detected by western blot using anti-HA and anti-FLAG antibodies. Source data is provided as a Source Data file.

(D) The percentage of crosslinking efficiency in panel S7C was calculated as mean+SD from 3 independent experiments. The highest increase in crosslink was observed when both *R1008I* and *W483R* were present. Source data is provided as a Source Data file.

(E) *In vivo* BMOE crosslink between *scc2 D369C* and *smc3-QQ K1004C* in the presence of *smc3 R1008I* or *W483R* or both using cells arrested in late G1. Scc1-PK was immunoprecipitated and co-immunoprecipitated Scc2-FLAG was detected by western blot using an anti-FLAG antibody. Source data is provided as a Source Data file.

(F) The distributions of *smc3-QQ_R1008I* suppressor mutations on Smc3 head. Source data is provided as a Source Data file.

(G) Tetrad diseection. Either Scc2 single mutation, *E822K* (green circles) or *L937F* (yellow circles), promotes cell proliferation of *smc3-QQ_R1008I* mutant (red circles). Source data is provided as a Source Data file.

(H) Recombinant tetramer of cohesin, Smc3 or $Smc3^{R1008I}$, Smc1, Scc1 and Scc3 was incubated with Scc2 or $Scc2^{E822K\_L937F}$. ATP was added to initiate the reaction, and the reaction rate was measured as the change in absorption at 360nm over time. Source data is provided as a Source Data file.

Supplementary Table 1 Cysteine pairs used in this study

<table>
<tr><th>Cysteine pair</th><th>Interface</th><th>Configuration</th><th>Purposes</th></tr>
<tr><td>Smc3 S1043C
Scc1 C56</td><td>Smc3 Coiled-coil/N-Scc1</td><td>Interaction between Smc3 Coiled-Coil/N-Scc1</td><td>To evaluate releasing activity by examining the association of N-Scc1/Smc3 CC</td></tr>
<tr><td>Smc1 N1192C
Smc3 R1222C</td><td>Cohesin heads</td><td>Engaged heads in E-cohesin</td><td>1. To examine ATP-mediated head engagement
2.To chemically stabilise engaged heads and E-cohesin</td></tr>
<tr><td>Smc1 R1031C
Smc3 E202C</td><td>Cohesin Coiled-Coils</td><td>Juxtaposed Coiled-Coils</td><td>To chemically stabilise juxtaposed CC and J-cohesin</td></tr>
<tr><td>Smc1 E1102C
Scc2 T1281C</td><td>Scc2/Smc1 head</td><td>Scc2/J-cohesin
Scc2/pre-E-cohesin
Scc2/E-cohesin</td><td>To examine effects of ATP binding and acetylation on Scc2/J-cohesin</td></tr>
<tr><td>Smc3 Q67C
Scc2 T1175C</td><td rowspan="2">Scc2/Smc3 head</td><td rowspan="2">Scc2/pre-E-cohesin</td><td rowspan="2">To examine Scc2/pre-E-cohesin interaction.</td></tr>
<tr><td>Smc3 M74C
Scc2 N886C</td></tr>
<tr><td>Smc3 S72C
Scc2 E819C</td><td rowspan="2">Scc2/Smc3 head</td><td rowspan="3">Scc2/E-cohesin</td><td rowspan="2">To examine the Scc2/Smc3 head interaction in Scc2/E-cohesin</td></tr>
<tr><td>Smc3 M74C
Scc2 E821C</td></tr>
<tr><td>Smc3 K1004C
Scc2 D369C</td><td>Scc2/Smc3 Coiled-Coil</td><td>To examine the Scc2/Smc3 CC interaction in Scc2/E-cohesin</td></tr>
</table>

Supplementary Table 2 Mutations used in this study

Mutations	Purposes
smc3-QQ	Mimicking Smc3 acetylation
smc3-K38I	Smc3 ATP-binding mutation, preventing ATP-mediated head engagement
smc3-E1155Q	Smc3 ATP-hydrolysis mutation, prolonging ATP-mediated head engagement
smc1-E1158Q	Smc1 ATP-hydrolysis mutation, prolonging ATP-mediated head engagement
smc3-R1008I	A mutation close to Smc3 Coiled Coil/Scc2 interface of Scc2/E-cohesin, partially suppressing the lethality of *smc3-QQ*
smc3-E199A	A mutation close to Smc3 Coiled Coil/Scc2 interface of Scc2/E-cohesin, partially suppressing the lethality of *smc3-QQ*
Smc3-W438R	A mutation close to Smc3 Coiled Coil elbow, with *R1008I*, greatly suppressing the lethality of *smc3-QQ*
scc2-EKLF	A hypermorphic Scc2 allele, with *R1008I*, greatly suppressing the lethality of *smc3-QQ*

Supplementary Table 3 yeast strains used in this study

Figures	Genotype	Origin	Strains
	All the S. cerevisiae strains derive from W303:, ade2-1, trp1-1, can1-100, leu2-3,112,his3-11,15, ura3, GAL, psi+	Nasmyth' lab	K699
**Figure 1 and S1**			
1B	*Mat a/alpha, Δsmc3::HIS3/Smc3, ura3::smc3(K112Q_K113Q)::URA3/ura3*	This study	B1356
	Mat a/alpha, Δsmc3::HIS3/Smc3, ura3::smc3(K112Q_K113Q_E199A)::URA3/ura3	This study	B1931
	Mat a/alpha, Δsmc3::HIS3/Smc3, ura3::smc3(K112Q_K113Q_R1008I)::URA3/ura3	This study	B1335
1D	*Mat a, trp1::SMC3-PK6::TRP1*	This study	K17407
	Mat a, trp1::smc3(K112Q_K113Q)-PK6::TRP1	This study	K22703
	Mat a, trp1::smc3(K112Q_K113Q_R1008I)-PK6::TRP1	This study	K22705
1F	*Mat a/alpha, Δsmc3::HIS3/SMC3, , ura3::smc3(K112Q_K113Q_R1008I_W483R)::URA3/ura3,, SCC1-PK9::KANMX/scc1*	This study	B1471
1G	*MATa, SCC1-PK9::KANMX*	This study	B910
	Mat a, SCC1-PK9::KANMX, Δsmc3::HIS3, ura3::smc3(K112Q_K113Q_R1008I)::URA3	This study	B2269
	Mat a, SCC1-PK9::KANMX, Δsmc3::HIS3, ura3::smc3(K112Q_K113Q_R1008I_W483R)::URA3	This study	B2851
1H	*MAT a/alpha, Δeco1::KANMX6/Eco1, smc3(W483R_R1008I)::HIS3/SMC3*	This study	B2902
	MAT a/alpha, Δeco1::KANMX6/Eco1, smc3(K112Q_K113Q_W483R_R1008I)::His3/SMC3	This study	B2903
1I	*ura3::smc3(S1043C)-PK6::URA3*	This study	B484
	ura3:smc3(K112Q, K113Q, S1043C)-PK6::URA3	This study	B483
	ura3::smc3(K112Q, K113Q, W483R, R1008I, S1043C)-PK6::URA3	This study	B4020
S1A	*MAT a*	Nasmyth	K699
	Mat a/alpha, Δsmc3::HIS3/SMC3, ura3::smc3(K112Q_K113Q_E199A)::URA3/ura3	This study	B1931
	Mat a/alpha, Δsmc3::HIS3/SMC3, ura3::smc3(K112Q_K113Q_R1008I)::URA3/ura3	This study	B1931
	Mat a, Δsmc3::HIS3, ura3::smc3(K112Q_K113Q_E199A)::URA3	This study	Dissected from B1931
	Mat a, Δsmc3::HIS3, ura3::smc3(K112Q_K113Q_R1008I)::URA3	This study	Dissected from B1931
**Figure 2 and S2**			

2C and S2A	*Mat a, smc1::NATMX4, SCC1-PK9::KANMX, Δmet15::smc1N1192C-myc9, ura3::smc3R1222C-HA3::URA3*	This study	B2936
	Mat a, smc1::NATMX4, SCC1-PK9::KANMX, Δmet15::smc1N1192C-myc9, ura3::smc3K112Q_K113Q_R1222C-HA3::URA3	This study	B2937
	Mat a, Δsmc1::NATMX4, SCC1-PK9::KanMX, Δmet15::smc1N1192C-myc9, ura3::smc3K38I_R1222C-HA3::URA3	This study	B3046
2E	*Mat a, SCC2_6xHis-FLAG6::KANMX*	This study	B1449
	Mat a, SCC2_6xHis-FLAG6::KANMX, ura3::SMC3-PK6::URA3	This study	B3026
	Mat a, SCC2_6xHis-FLAG6::KANMX, ura3::smc3(K112Q_K113Q)-PK6::URA3	This study	B3027
**Figure 3 and S3**			
3B	*MATa, SCC1-PK9::KANMX, SCC2_6xHis-FLAG6::KANMX, pBH760 (smc3-L111TAG-HA3), pBH61 (tRNATyr/Bpa synthetase)*	This study	B1852
	MATa, SCC1-PK9::KANMX, SCC2_6xHis-FLAG6::KANMX, pBH761 (smc3-Q117TAG-HA3), pBH61 (tRNATyr/Bpa synthetase)	This study	B1855
	MATa, SCC1-PK9::KANMX, SCC2_6xHis-FLAG6::KANMX, pBH758 (smc3-K57TAG-HA3), pBH61 (tRNATyr/Bpa synthetase)	This study	B1850
	MATa, SCC1-PK9::KANMX, SCC2_6xHis_FLAG6::KANMX, pBH796 (smc3-R58TAG-HA3), pBH61 (tRNATyr/Bpa synthetase)	This study	B1900
	MATa, SCC1-PK9::KANMX, SCC2_6xHis_FLAG6::KANMX, pBH773 (smc3-R61TAG-HA3), pBH61 (tRNATyr/Bpa synthetase)	This study	B1848
	MATa, SCC1-PK9::KANMX, SCC2_6xHis_FLAG6::KANMX, pBH759 (smc3-M74TAG-HA3), pBH61 (tRNATyr/Bpa synthetase)	This study	B1854
	MATa, SCC1-PK9::KANMX, SCC2_6xHis_FLAG6::KANMX, pBH787 (Smc3-H66TAG-HA3), pBH61 (tRNATyr/Bpa synthetase)	This study	B1878
	MATa, SCC1-PK9::KANMX, SCC2_6xHis_FLAG6::KANMX, pBH585 (smc3-Q67TAG-HA3), pBH61 (tRNATyr/Bpa synthetase)	This study	B1505
3D	*MATa, SCC1-PK9::KANMX, SCC2_6xHis_FLAG6::KANMX, pBH1059 (smc1D1069TAG-myc9), pBH61 (tRNATyr/Bpa synthetase)*	This study	B3201
	MATa, SCC1-PK9::KANMX, SCC2_6xHis_FLAG6::KANMX, pBH1060 (smc1D1073TAG-myc9), pBH61 (tRNATyr/Bpa synthetase)	This study	B3202
	MATa, SCC1-PK9::KANMX, SCC2_6xHis_FLAG6::KANMX, pBH1061	This study	B3203

	(smc1D1076TAG-myc9), pBH61 (tRNATyr/Bpa synthetase)		
	MATa, SCC1-PK9::KANMX, SCC2_6xHis_FLAG6::KANMX, pBH1062 (smc1R1080TAG-myc9), pBH61 (tRNATyr/Bpa synthetase)	This study	B3204
	MATa, SCC1-PK9::KANMX, SCC2_6xHis_FLAG6::KANMX, pBH1055 (smc1T1100TAG-myc9), pBH61 (tRNATyr/Bpa synthetase)	This study	B3199
	MATa, SCC1-PK9::KANMX, SCC2_6xHis_FLAG6::KANMX, pBH1056 (smc1E1102TAG-Myc9), pBH61 (tRNATyr/Bpa synthetase)	This study	B2814
	MATa, Scc1-PK9::KANMX, SCC2_6xHis_FLAG6::KANMX, pBH1057 (smc1K1113TAG-Myc9), pBH61 (tRNATyr/Bpa synthetase)	This study	B2815
	MATa, SCC1-PK9::KANMX, SCC2_6xHis_FLAG6::KANMX, pBH1058 (smc1T1117TAG-myc9), pBH61 (tRNATyr/Bpa synthetase)	This study	B3200
	MATa, SCC1-PK9::KANMX, SCC2_6xHis_FLAG6::KANMX, pBH1047 (smc1L1120TAG-Myc9), pBH61 (tRNATyr/Bpa synthetase)	This study	B3193
	MATa, SCC1-PK9::KANMX, SCC2_6xHis_FLAG6::KANMX, pBH1048 (smc1R1122TAG-Myc9), pBH61 (tRNATyr/Bpa synthetase)	This study	B2811
	MATa, SCC1-PK9::KANMX, SCC2_6xHis_FLAG6::KANMX, pBH1049 (smc1F1123TAG-myc9), pBH61 (tRNATyr/Bpa synthetase)	This study	B3196
	MATa, SCC1-PK9::KANMX, SCC2_6xHis_FLAG6::KANMX, pBH1050 (smc1K1124TAG-Myc9), pBH61 (tRNATyr/Bpa synthetase)	This study	B2812
	MATa, SCC1-PK9::KANMX, SCC2_6xHis_FLAG6::KANMX, pBH1051 (smc1E1127TAG-Myc9), pBH61 (tRNATyr/Bpa synthetase)	This study	B2813
	MATa, SCC1-PK9::KANMX, SCC2_6xHis_FLAG6::KANMX, pBH1052 (smc1Y1128TAG-myc9), pBH61 (tRNATyr/Bpa synthetase)	This study	B3197
3E	*MATa, SCC1-PK9::KANMX, Δscc2::NATMX4, smc3HA6::HIS3, Δlys2:: SCC2_6xHis_FLAG6*	This study	B3161
	MATa, SCC1-PK9::KANMX, Δscc2::NATMX4, smc3HA6::HIS3, Δlys2::scc2T1175C_HIS6_FLAG6	This study	B3176

	MATa, SCC1-PK9::KANMX, Δscc2::NATMX4, smc3_Q67C_HA::His3, Δlys2:: SCC2_6xHis_FLAG6	This study	B3054
	MATa, , SCC1-PK9::KANMX, Δscc22::NATMX4, smc3_Q67C_HA::His3, Δlys2::scc2T1175C_HIS6_FLAG6	This study	B3064
3F	*MATa,smc1-myc9:: kiTRP1, SCC1-PK9::KANMX, Δscc2::NATMX4, Δlys2:: SCC2_6xHis_FLAG6*	This study	B3115
	MATa, smc1-myc9::kiTRP1, SCC1-PK9::KANMX, Δscc2::NATMX4, Δlys2:: scc2T1281C_HIS6_FLAG6,	This study	B3110
	MATa, SCC1-PK9::KANMX, Δscc2::NATMX4, smc1E1102Cmyc9:: kiTRP1, Δlys2:: SCC2_6xHis_FLAG6	This study	B3097
	MATa, SCC1-PK9::KANMX, Δscc2::NATMX4, smc1E1102Cmyc9:: kiTRP1, Δlys2::scc2T1281C_HIS6_FLAG6	This study	B3043
3G	*MATa, , SCC1-PK9::KANMX, Δscc22::NATMX4, smc3_Q67C_HA::His3, Δlys2::scc2T1175C_HIS6_FLAG6*	This study	B3064
	MATa, SCC1-PK9::KANMX, Δscc2::NATMX4, smc1E1102Cmyc9:: kiTRP1, Δlys2::scc2T1281C_HIS6_FLAG6	This study	B3043
	MATa, SCC1-PK9::KANMX, Δscc2::NATMX4, smc3Q67C_HA6::spHis5, Δlys2::Scc2T1281C_HIS6_FLAG6	This study	B3174
	MATa, SCC1-PK9::KANMX, ΔSCC2::NATMX4, smc1E1102Cmyc9:: kiTRP1, Δlys2:: scc2T1175C_HIS6_FLAG6	This study	B3172
	MATa, SCC1-PK9::KANMX, Δscc2::NATMX4, smc3Q67C_HA6::spHis5, smc1E1102Cmyc9:: kiTRP1, Δlys2:: scc2_T1175C_T1281C_HIS6_FLAG6	This study	B3194
S3A and S3B	*Mat a, SCC1-PK9::KanMX, pBH585 (smc3-Q67TAG-HA3), pBH61 (tRNATyr/Bpa synthetase)*	This study	B1613
	Mat a, SCC1-PK9::KanMX, SCC2_6xHis_FLAG6::KANMX, pBH585 (smc3-Q67TAG-HA3), pBH61 (tRNATyr/Bpa synthetase)	This study	B1505
	Mat a, SCC1-PK9::KanMX, Δsmc1::NatMX4, URA3::Smc1-Myc9::URA3, pBH585 (Smc3-Q67TAG-HA3), pBH61 (tRNATyr/Bpa synthetase)	This study	B1614
S3C	*MATa, SCC1-PK9::KANMX, pBH1056 (smc1E1102TAG-Myc9), pBH61 (tRNATyr/Bpa synthetase)*	This study	B2797
	*MATa, SCC1-PK9::KANMX, SCC2_6xHis_FLAG6::KANMX, pBH1056 (smc1E1102TAG-Myc9), pBH61 (*tRNA^{Tyr}/Bpa synthetase*)*	This study	B2814

	Mat a, SMC3-GFP::URA3, SCC1-PK9::KANMX, pBH1056(smc1E1102TAG-Myc9 in YEplac181), pBH61 (tRNATyr/Bpa synthetase)	This study	B2980
	Mat a, SMC3-GFP::URA3, SCC1-PK9::KANMX, SCC2_6xHis_FLAG6::KANMX, pBH1056(smc1E1102TAG-Myc9 in YEplac181), pBH61 (tRNATyr/Bpa synthetase)	This study	B3028
S3D	*MATa, SCC1-PK9::KANMX, SCC2_6xHis_FLAG6::KANMX, pBH1056 (smc1E1102TAG-Myc9), pBH61 (tRNATyr/Bpa synthetase)*	This study	B2814
	MAT a, SCC1-PK9::KANMX, scc2TEV215_6xHis_FLAG6:: KANMX, pBH61 (tRNATyr/Bpa synthetase), pBH585 (smc3-Q67TAG-HA3)	This study	B2171
	MAT a, SCC1-PK9::KANMX, scc2TEV471_6xHis_FLAG6:: KANMX, pBH61 (tRNATyr/Bpa synthetase), pBH585 (smc3-Q67TAG-HA3)	This study	B2172
	MAT a, SCC1-PK9::KANMX, scc2TEV668_6xHis_FLAG6:: KANMX, pBH61 (tRNATyr/Bpa synthetase), pBH585 (smc3-Q67TAG-HA3)	This study	B2145
	MAT a, SCC1-PK9::KANMX, scc2TEV843_6xHis_FLAG6:: KANMX, pBH61 (tRNATyr/Bpa synthetase), pBH585 (smc3-Q67TAG-HA3)	This study	B2149
	MAT a, SCC1-PK9::KANMX, scc2TEV888_6xHis_FLAG6:: KANMX, pBH61 (tRNATyr/Bpa synthetase), pBH585 (smc3-Q67TAG-HA3)	This study	B2152
	MAT a, SCC1-PK9::KANMX, scc2TEV917_6xHis_FLAG6:: KANMX, pBH61 (tRNATyr/Bpa synthetase), pBH585 (smc3-Q67TAG-HA3)	This study	B2147
	MAT a, SCC1-PK9::KANMX, scc2TEV1176_6xHis_FLAG6:: KANMX, pBH61 (tRNATyr/Bpa synthetase), pBH585 (smc3-Q67TAG-HA3)	This study	B2170
	MAT a, SCC1-PK9::KANMX, scc2TEV1222_6xHis_FLAG6:: KANMX, pBH61 (tRNATyr/Bpa synthetase), pBH585 (smc3-Q67TAG-HA3)	This study	B2151
	MAT alpha, SCC1-PK9::KANMX, scc2TEV1053_6xHis_FLAG6:: KANMX, pBH61 (tRNATyr/Bpa synthetase), pBH585 (smc3-Q67TAG-HA3)	This study	B2767
	MAT a, SCC1-PK9::KANMX, scc2TEV1077_6xHis_FLAG6:: KANMX, pBH61	This study	B2768

	(tRNATyr/Bpa synthetase), pBH585 (smc3-Q67TAG-HA3)		
	MAT a SCC1-PK9::KANMX, scc2TEV1109_6xHis_FLAG6:: KANMX, pBH61 (tRNATyr/Bpa synthetase), pBH585 (smc3-Q67TAG-HA3)	This study	B2769
S3E	*MATa, SCC1-PK9::KANMX, Δscc2::NATMX4, smc3_Q67C_HA6::spHis5, Δlys2:: scc2T1110C_HIS6_FLAG6*	This study	B3050
	MATa, SCC1-PK9::KANMX, Δscc2::NATMX4, smc3_Q67C_HA6::spHis5, Δlys2:: scc2K11300C_HIS6_FLAG6	This study	B3080
	MATa, SCC1-PK9::KANMX, Δscc2::NATMX4,smc3_Q67C_HA6::spHis5, Δlys2:: scc2R1135C_HIS6_FLAG6	This study	B3062
	MATa, SCC1-PK9::KANMX, Δscc2::NATMX4, smc3_Q67C_HA6::spHis5, Δlys2:: scc2Q1146C_HIS6_FLAG6	This study	B3051
	MATa, SCC1-PK9::KANMX, Δscc2::NATMX4, smc3_Q67C_HA6::spHis5, Δlys2:: scc2D1148C_HIS6_FLAG6	This study	B3061
	MATa, SCC1-PK9::KANMX, Δscc2::NATMX4, smc3_Q67C_HA6::spHis5, Δlys2:: scc2L1150C_HIS6_FLAG6	This study	B3052
	MATa, SCC1-PK9::KANMX, Δscc2::NATMX4, smc3_Q67C_HA6::spHis5, Δlys2:: scc2D1151C_HIS6_FLAG6	This study	B3063
	MATa, SCC1-PK9::KANMX, Δscc2::NATMX4, smc3_Q67C_HA6::spHis5, Δlys2:: scc2S1171C_HIS6_FLAG6	This study	B3053
	MATa, SCC1-PK9::KANMX, Δscc2::NATMX4, smc3_Q67C_HA6::spHis5, Δlys2:: scc2T1175C_HIS6_FLAG6	This study	B3064
	MATa, SCC1-PK9::KANMX, Δscc2::NATMX4, smc3_Q67C_HA6::spHis5, Δlys2::SCC2_HIS6_FLAG6	This study	B3054
S3F	*MATa, SCC1-PK9::KANMX, SCC2_6xHis_FLAG6::KANMX, pBH1056 (smc1E1102TAG-Myc9), pBH61 (tRNATyr/Bpa synthetase)*	This study	B2814
	Mat a, SCC1-PK9::KANMX, scc2TEV215_6xHis_FLAG6:: KANMX, pBH1056(smc1E1102TAG-Myc9 in YEplac181), pBH61 (tRNATyr/Bpa synthetase)	This study	B2906
	Mat a, SCC1-PK9::KANMX, scc2TEV471_6xHis_FLAG6:: KANMX, pBH1056(smc1E1102TAG-Myc9 in YEplac181), pBH61 (tRNATyr/Bpa synthetase)	This study	B2907

	Mat a, SCC1-PK9::KANMX, scc2TEV668_6xHis_FLAG6:: KANMX, pBH1056(smc1E1102TAG-Myc9 in YEplac181), pBH61 (tRNATyr/Bpa synthetase)	This study	B2908
	Mat a, SCC1-PK9::KANMX, scc2TEV843_6xHis_FLAG6:: KANMX, pBH1056(smc1E1102TAG-Myc9 in YEplac181), pBH61 (tRNATyr/Bpa synthetase)	This study	B2909
	Mat a, SCC1-PK9::KANMX, scc2TEV1053_6xHis_FLAG6:: KANMX, pBH1056(smc1E1102TAG-Myc9 in YEplac181), pBH61 (tRNATyr/Bpa synthetase)	This study	B2911
	Mat a, SCC1-PK9::KANMX, scc2TEV1222_6xHis_FLAG6:: KANMX, pBH1056(smc1E1102TAG-Myc9 in YEplac181), pBH61 (tRNATyr/Bpa synthetase)	This study	B2910
	Mat a, SCC1-PK9::KANMX, scc2TEV1260_6xHis_FLAG6:: KANMX, pBH1056(smc1E1102TAG-Myc9 in YEplac181), pBH61 (tRNATyr/Bpa synthetase)	This study	B3017
	Mat a, SCC1-PK9::KANMX, scc2TEV1303_6xHis_FLAG6:: KANMX, pBH1056(smc1E1102TAG-Myc9 in YEplac181), pBH61 (tRNATyr/Bpa synthetase)	This study	B3019
	Mat a, SCC1-PK9::KANMX, scc2TEV1351_6xHis_FLAG6:: KANMX, pBH1056(smc1E1102TAG-Myc9 in YEplac181), pBH61 (tRNATyr/Bpa synthetase)	This study	B3015
	Mat a, SCC1-PK9::KANMX, scc2TEV1405_6xHis_FLAG6:: KANMX, pBH1056(smc1E1102TAG-Myc9 in YEplac181), pBH61 (tRNATyr/Bpa synthetase)	This study	B3016
	Mat a, SCC1-PK9::KANMX, scc2TEV1456_6xHis_FLAG6:: KANMX, pBH1056(smc1E1102TAG-Myc9 in YEplac181), pBH61 (tRNATyr/Bpa synthetase)	This study	B3018
S3G	*MATa, SCC1-PK9::KANMX, scc2::NATMX4, smc1E1102Cmyc9:: kiTRP1, Δlys2:: scc2L1225C_HIS6_FLAG6*	This study	B3034
	MATa, SCC1-PK9::KANMX, scc2::NATMX4, smc1E1102Cmyc9:: kiTRP1, Δlys2:: scc2N1245C_HIS6_FLAG6	This study	B3035
	MATa, SCC1-PK9::KANMX, scc2::NATMX4, smc1E1102Cmyc9:: kiTRP1, Δlys2:: scc2L1261C_HIS6_FLAG6	This study	B3036
	MATa, SCC1-PK9::KANMX, scc2::NATMX4, smc1E1102Cmyc9:: kiTRP1, Δlys2:: scc2L1277C_HIS6_FLAG6	This study	B3042

	MATa, SCC1-PK9::KANMX, scc2::NATMX4, smc1E1102Cmyc9:: kiTRP1, Δlys2:: scc2T1281C_HIS6_FLAG6	This study	B3043
	MATa, SCC1-PK9::KANMX, scc2::NATMX4, smc1E1102Cmyc9:: kiTRP11, Δlys2:: scc2R1289C_HIS6_FLAG6	This study	B3037
	MATa, SCC1-PK9::KANMX, scc2::NATMX4, smc1E1102Cmyc9:: kiTRP1, Δlys2:: scc2L1302C_HIS6_FLAG6	This study	B3037
	MATa, SCC1-PK9::KANMX, scc2::NATMX4, smc1E1102Cmyc9:: kiTRP1, Δlys2:: scc2L1307C_HIS6_FLAG6	This study	B3038
	MATa, SCC1-PK9::KANMX, scc2::NATMX4, smc1E1102Cmyc9:: kiTRP1, Δlys2:: scc2L1315C_HIS6_FLAG6	This study	B3040
	MATa, SCC1-PK9::KANMX, scc2::NATMX4, smc1E1102Cmyc9:: kiTRP1, Δlys2:: scc2L1325C_HIS6_FLAG6	This study	B3041
	MATa, SCC1-PK9::KANMX, scc2::NATMX4, smc1E1102Cmyc9:: kiTRP1, Δlys2:: scc2Y1279C_HIS6_FLAG6	This study	B3074
	MATa, SCC1-PK9::KANMX, scc2::NATMX4, smc1E1102Cmyc9:: kiTRP1, Δlys2:: scc2L1280C_HIS6_FLAG6	This study	B3075
	MATa, SCC1-PK9::KANMX, scc2::NATMX4, smc1E1102Cmyc9:: kiTRP1, Δlys2:: scc2V1283C_HIS6_FLAG6	This study	B3076
	MATa, SCC1-PK9::KANMX, scc2::NATMX4, smc1E1102Cmyc9:: kiTRP1, Δlys2:: scc2S1285C_HIS6_FLAG6	This study	B3077
	MATa, SCC1-PK9::KANMX, scc2::NATMX4, smc1E1102Cmyc9:: kiTRP1, Δlys2:: scc2L1287C_HIS6_FLAG6	This study	B3078
	**Figure 4 and S4**		
4B and S4E	*Mat a,Δsmc3::HIS3, SCC1-PK9::KANMX, Δmet15::smc3M74C_HA3, Δscc2::NATMX4, Δlys2:: scc2N780C_HIS6_FLAG6*	This study	B3274
	Mat a, Δsmc3::HIS3, SCC1-PK9::KANMX, Δmet15::smc3M74C_HA3, Δscc2::NATMX4, Δlys2:: scc2D782C_HIS6_FLAG6	This study	B3276
	Mat a, Δsmc3::HIS3, Δmet15::smc3M74C_HA3, SCC1-PK9::KANMX, Δscc2::NATMX4, Δlys2:: scc2E819C_HIS6_FLAG6	This study	B3272
	Mat a, SCC1-PK9::KANMX, Δsmc3::HIS3, Δmet15::sm3M74C_HA3, Δscc2::NATMX4, Δlys2:: scc2D820C_HIS6_FLAG6	This study	B3268
	Mat a, SCC1-PK9::KANMX, Δsmc3::HIS3, Δmet15::smc3M74C_HA3, Δscc2::NATMX4, Δlys2:: scc2E821C_HIS6_FLAG6	This study	B3323

	Mat a, Δscc2::NATMX4, Δsmc3::HIS3, SCC1-PK9::KANMX, Δmet15::smc3M74C_HA3, Δlys2::Scc2R830C_HIS6_FLAG6	This study	B3432
	Mat a, Δscc2::NATMX4, Δsmc3::HIS3, SCC1-PK9::KANMX, Δmet15::smc3M74C_HA3, Δlys2:: scc2L878C_HIS6_FLAG6	This study	B3433
	Mat a, Δscc2::NATMX4, Δsmc3::HIS3, SCC1-PK9::KANMX, Δmet15::smc3M74C_HA3, Δlys2:: scc2N886C_HIS6_FLAG6	This study	B3434
4C and S4H,	*Mat a, smc3E202C-HA6::spHis5, SCC1-PK9:: NATMX, SCC2_6xHis_FLAG6::KANMX,pBH1194 (smc1(R1031C_E1102TAG)-myc), pBH61 (tRNATyr/Bpa synthetase)*	This study,	B3227
	Mat a, SCC1-PK9::KANMX, Δsmc1::NATMX4, Δmet15::smc1R1031C_Myc9, SCC2_6xHis_FLAG6::KANMX, pBH1092(smc3E202C_Q67TAG-HA3), pBH61 (tRNATyr/Bpa synthetase)	This study,	B2285
4D and S4I	*Mat a, SCC1-PK9::KANMX, Δsmc1::NATMX4, Δmet15::smc1N1192C_Myc9, SCC2_6xHis_FLAG6::KANMX, pBH1092(smc3R1222C_Q67TAG-HA3),pBH61 (tRNATyr/Bpa synthetase)*	This study	B3009
	Mat a, SCC1-PK9::NATMX, smc3R1222C_HA6::SpHis3, SCC2_6xHis_FLAG6::KANMX, pBH1121 (smc1N1192C_E1102TAG-myc9), pBH61 (tRNATyr/Bpa synthetase)	This study	B4041
S4G	*MAT a, Δsmc1::NATMX4, SCC1-PK9::KANMX Δmet15::smc1K191CMyc9,Δsmc3::HIS3, ura3::smc3K184C-HA3 (pBH986)::URA3*	This study	B2656
	Mat alpha, Δsmc1::NATMX4, SCC1-PK9::KANMX, Δmet15::smc1K191CMyc9 , Δsmc3::HIS3, ura3::smc3E188C-HA3::URA3	This study	B2612
	MAT a, SCC1-PK9::KANMX smc3Q195C-HA6::His3MX6, Δsmc1::NATMX4, leu2::smc1S195C-myc9::HPHNT1	This study	B2423
	Mat a, SCC1-PK9::KANMX Δsmc1::NATMX4, leu2::smc1K201C-myc9::HPHNT1, smc3K198C-HA6::His3MX6	This study	B2395
	Mat a, Δsmc1::NATMX4, Smc3E202C-HA6::spHis5, SCC1-PK9::KANMX Δmet15:sSmc1E209C-myc9	This study	B2311
	Mat a, Δsmc1::NATMX4, smc3E202C-HA6::spHis5, SCC1-PK9::KANMX Δmet15::smc1R1031C-myc9	This study	B2233
	Mat a, Δsmc1::NATMX4, SCC1-PK9::KANMX Δmet15::smc1R1020C-myc9, smc3E213C-HA6::His3MX6	This study	B2376

	Figure 5 and S5		
5B and S5B	*Mat a, SCC1-PK9::NATMX, Scc2_6xHis_FLAG6::KANMX, Δsmc3::HIS3, Δmet15::Smc3_HA3*	This study	B3476
	Mat a, SCC1-PK9::NATMX, scc2E819C_HIS6_FLAG6::KANMX, Δsmc3::HIS3, Δmet15::Smc3_HA3	This study	B3475
	Mat a, Δsmc3::HIS3, SCC1-PK9::NATMX, Δmet15::smc3S72C_HA3, SCC2_6xHis_FLAG6::KANMX	This study	B3473
	Mat a, Δsmc3::HIS3, SCC1-PK9::NATMX, Δmet15::smc3S72C_HA3, scc2E819C_HIS6_FLAG6::KANMX	This study	B3471
5C and S5C	*MATa,SCC1-PK9::NATMX, SMC3HA6::HIS3, SCC2_6xHis_FLAG6::KANMX, leu2::Gal1p-Sic1(9m)/His3p-Gal1/His3p-Gal2/Gal1p-Gal4::Leu2*	This study	B3913
	MATa, SCC1-PK9::NATMX, scc2_D369C_FLAG6::KanMX, SMC3-HA6::HIS3, leu2::Gal1p-Sic1(9m)/His3p-Gal1/His3p-Gal2/Gal1p-Gal4::Leu2	This study	B3929
	MATa,SCC1-PK9::NATMX, smc3_K1004C_HA6::His3, SCC2_6xHis_FLAG6::KANMX, leu2::Gal1p-Sic1(9m)/His3p-Gal1/His3p-Gal2/Gal1p-Gal4::Leu2	This study	B3927
	MATa, SCC1-PK9::NATMX, Smc3_K1004C_HA6::His3, scc2_D369C_FLAG6::KanMX, leu2::Gal1p-Sic1(9m)/His3p-Gal1/His3p-Gal2/Gal1p-Gal4::Leu2	This study	B3916
5D and 5D	*Mat a, SCC1-PK6::TRP1, ura3::smc3Q67C-HA3::URA3, Scc2T1175C_HIS6_FLAG6::KANMX, leu2::Gal1p-Sic1(9m)/His3p-Gal1/His3p-Gal2/Gal1p-Gal4::Leu2*	This study	B3819
	Mat a, SCC1-PK6::TRP1, ura3::smc3Q67C_E1155Q-HA3::URA3, scc2T1175C_HIS6_FLAG6::KANMX, leu2::Gal1p-Sic1(9m)/His3p-Gal1/His3p-Gal2/Gal1p-Gal4::Leu2	This study	B3820
	MATa, SCC1-PK9::NATMX, ura3::smc3_S72C_HA3::URA3, scc2E819C_HIS6_FLAG6::KANMX, leu2::Gal1p-Sic1(9m)/His3p-Gal1/His3p-Gal2/Gal1p-Gal4::Leu2	This study	B3821
	MATa, SCC1-PK9::NATMX, ura3::smc3_S72C_E1155Q_HA3::URA3, scc2E819C_HIS6_FLAG6::KANMX, leu2::Gal1p-Sic1(9m)/His3p-Gal1/His3p-Gal2/Gal1p-Gal4::Leu2	This study	B3822

	MATa, SCC1-PK9::NATMX, ura3::smc3_K1004C_HA3::URA3, scc2E819C_HIS6_FLAG6::KANMX, leu2::Gal1p-Sic1(9m)/His3p-Gal1/His3p-Gal2/Gal1p-Gal4::Leu2	This study	B3880
	MATa, SCC1-PK9::NATMX, ura3::smc3_K1004C_E1155Q_HA3::URA3, scc2E819C_HIS6_FLAG6::KANMX, leu2::Gal1p-Sic1(9m)/His3p-Gal1/His3p-Gal2/Gal1p-Gal4::Leu2	This study	B3890
	MATa, SCC1-PK9::NATMX, ura3::smc1E1102C-MYC9::URA3, scc2T1281C_HIS6_FLAG6::KANMX, leu2::Gal1p-Sic1(9m)/His3p-Gal1/His3p-Gal2/Gal1p-Gal4::Leu2	This study	B3823
	MATa, SCC1-PK9::NATMX, ura3::smc1E1102C_E1158Q-MYC9::URA3, scc2T1281C_HIS6_FLAG6::KANMX, leu2::Gal1p-Sic1(9m)/His3p-Gal1/His3p-Gal2/Gal1p-Gal4::Leu2	This study	B3824
5E	*MATa, SCC1-PK9::HYGMX, Ura3::smc3_S72C_K1004C-HA3::URA3, scc2D369C_E819C_HIS6_FLAG6::KANMX, leu2::Gal1p-Sic1(9m)/His3p-Gal1/His3p-Gal2/Gal1p-Gal4::Leu2*	This study	B3892
	MATa, SCC1-PK9::HYGMX, Ura3::smc3_K1004C_E1155Q-HA3::URA3, scc2D369C_HIS6_FLAG6::KANMX, leu2::Gal1p-Sic1(9m)/His3p-Gal1/His3p-Gal2/Gal1p-Gal4::Leu2	This study	B3890
	MATa, SCC1-PK9::HYGMX, Ura3::smc3_S72C_E1155Q-HA3::URA3, scc2D369C_E819C_HIS6_FLAG6::KANMX, leu2::Gal1p-Sic1(9m)/His3p-Gal1/His3p-Gal2/Gal1p-Gal4::Leu2	This study	B3822
	MATa, SCC1-PK9::HYGMX, Ura3::smc3_S72C_K1004C-E1155Q-HA3::URA3, scc2D369C_E819C_HIS6_FLAG6::KANMX, leu2::Gal1p-Sic1(9m)/His3p-Gal1/His3p-Gal2/Gal1p-Gal4::Leu2	This study	B3891
5F	*MATa, SCC1-PK9::HYGMX, Ura3::smc3_K1004C_E1155Q-HA3::URA3, scc2D369C_HIS6_FLAG6::KANMX, leu2::Gal1p-Sic1(9m)/His3p-Gal1/His3p-Gal2/Gal1p-Gal4::Leu2*	This study	B3890
	MATa, SCC1-PK9::HYGMX, Ura3::smc3_S72C_E1155Q-HA3::URA3, scc2D369C_E819C_HIS6_FLAG6::KANMX, leu2::Gal1p-Sic1(9m)/His3p-Gal1/His3p-Gal2/Gal1p-Gal4::Leu2	This study	B3822

	MATa, SCC1-PK9::HYGMX, Ura3::smc3_S72C_K1004C-E1155Q-HA3::URA3, scc2D369C_E819C_HIS6_FLAG6::KANMX, leu2::Gal1p-Sic1(9m)/His3p-Gal1/His3p-Gal2/Gal1p-Gal4::Leu2	This study	B3891
	MATa, SCC1-PK9::HYGMX, Ura3::smc3_Q67C_E1155Q-HA3::URA3, scc2 T1175C_HIS6_FLAG6::KANMX, leu2::Gal1p-Sic1(9m)/His3p-Gal1/His3p-Gal2/Gal1p-Gal4::Leu2	This study	B4063
	MATa, SCC1-PK9::HYGMX, Ura3::smc3_Q67C_K1004C-E1155Q-HA3::URA3, scc2D369C_T1175C_HIS6_FLAG6::KANMX, leu2::Gal1p-Sic1(9m)/His3p-Gal1/His3p-Gal2/Gal1p-Gal4::Leu2	This study	B4066
	**Figure 6 and S6**		
6A and S56	*MATa, SCC1-PK9::KANMX, Δscc2::NATMX4, Δlys2:: scc2T1175C_HIS6_FLAG6, Δmet15::smc3Q67C-HA3, leu2::Gal1p-Sic1(9m)/His3p-Gal1/His3p-Gal2/Gal1p-Gal4::Leu2*	This study	B3262
	MATa, SCC1-PK9::KANMX, Δscc2::NATMX4, Δlys2:: scc2T1175C_HIS6_FLAG6, Δmet15:: smc3(K112Q_K113Q_Q67C) -HA3, leu2::Gal1p-Sic1(9m)/His3p-Gal1/His3p-Gal2/Gal1p-Gal4::Leu2	This study	B3263
6C and S6C	*Mat a, SCC1-PK9::KANMX, Δmet15:smc3S72C_HA3, Δscc2::NATMX4, Δlys2::scc2E819C_HIS6_FLAG6, leu2::Gal1p-Sic1(9m)/His3p-Gal1/His3p-Gal2/Gal1p-Gal4::Leu2*	This study	B3371
	Mat a, SCC1-PK9::KANMX, Δmet15::smc3(K112Q_K113Q_S72C)-HA3, Δscc2::NATMX4, Δlys2::scc2E819C_HIS6_FLAG6, leu2::Gal1p-Sic1(9m)/His3p-Gal1/His3p-Gal2/Gal1p-Gal4::Leu2	This study	B3417
6E and S6E	*MATa, SCC1-PK6::TRP1, Ura3::smc3_K1004C_HA3::URA3, scc2_D369C_FLAG6::KanMx, leu2::Gal1p-Sic1(9m)/His3p-Gal1/His3p-Gal2/Gal1p-Gal4::Leu2*	This study	B3814
	MATa,SCC1-PK6::TRP1, Ura3::smc3_(K112Q_K113Q_K1004C)-HA3::URA3, scc2_D369C_FLAG6::KanMx, leu2::Gal1p-Sic1(9m)/His3p-Gal1/His3p-Gal2/Gal1p-Gal4::Leu2	This study	B3815
5G	*MATa, Δscc2::NATMX4, smc1E1102Cmyc9:: kiTRP1, Δlys2:: scc2T1281C_HIS6_FLAG6, ura3::smc3(K112Q, K113Q)-PK6::URA3 ,*	This study	B3265

	leu2::Gal1p-Sic1(9m)/His3p-Gal1/His3p-Gal2/Gal1p-Gal4::Leu2		
	MATa, Δscc2::NATMX4, smc1E1102Cmyc9::kiTRP1, Δlys2:: scc2T1281C_HIS6_FLAG6, ura3::SMC3-PK6::URA3 , leu2::Gal1p-Sic1(9m)/His3p-Gal1/His3p-Gal2/Gal1p-Gal4::Leu2	This study	B3266
	MATa, Δscc2::NATMX4, smc1E1102Cmyc9::kiTRP1, Δlys2:: scc2T1281C_HIS6_FLAG6, leu2::Gal1p-Sic1(9m)/His3p-Gal1/His3p-Gal2/Gal1p-Gal4::Leu2	This study	B3267
S6B	*MATa, SCC1-PK9::KANMX, SCC2_6xHis_FLAG6::KANMX, pBH585 (smc3-Q67TAG-HA3), pBH61 (tRNATyr/Bpa synthetase)*	This study	B1505
	MATa, SCC1-PK9::KANMX, SCC2_6xHis_FLAG6::KANMX, pBH323 (smc3QQ-Q67TAG-HA3), pBH61 (tRNATyr/Bpa synthetase)	This study	B2762
S6C	*Mat a, Δbar1::hisG Scc1-PK6::TRP1 Scc2T1175C_HIS6_FLAG6::KANMX Δmet15::Smc3Q67C-HA3 his3::ADH1 promoter-OsTIR1-9myc::HIS3 Pds5-AID*–9myc::KanMX leu2::Gal1p-Sic1(9m)/His3p-Gal1/His3p-Gal2/Gal1p-Gal4::Leu2*	This study	B4257
	Mat a, Δbar1::hisG Scc1-PK6::TRP1 Scc2T1175C_HIS6_FLAG6::KANMX Δmet15::Smc3QQ_Q67C-HA3 his3::ADH1 promoter-OsTIR1-9myc::HIS3 Pds5-AID–9myc::KanMX leu2::Gal1p-Sic1(9m)/His3p-Gal1/His3p-Gal2/Gal1p-Gal4::Leu2*	This study	B4259
	**Figure 7 and S7**		
7A and S7A	*MATa, , SCC1-PK9::KANMX, Δscc2::NATMX4, Δlys2::scc2T1175C_HIS6_FLAG6, Δmet15::smc3Q67C-HA3, leu2::Gal1p-Sic1(9m)/His3p-Gal1/His3p-Gal2/Gal1p-Gal4::Leu2*	This study	B3262
	MATa, , SCC1-PK9::KANMX, Δscc2::NATMX4, Δlys2:scc2T1175C_HIS6_FLAG6, Δmet15::smc3(K112Q_K113Q_Q67C)-HA3, leu2::Gal1p-Sic1(9m)/His3p-Gal1/His3p-Gal2/Gal1p-Gal4::Leu2	This study	B3263
	MATa, , SCC1-PK9::KANMX, Δscc2::NATMX4, Δlys2:scc2T1175C_HIS6_FLAG6, Δmet15::smc3(K112Q_K113Q_Q67C-R1008I)-HA3, leu2::Gal1p-Sic1(9m)/His3p-Gal1/His3p-Gal2/Gal1p-Gal4::Leu2	This study	B3362

	MATa, , SCC1-PK9::KANMX, Δscc2::NATMX4, Δlys2:scc2T1175C_HIS6_FLAG6, Δmet15::smc3(K112Q_K113Q_Q67C-W483R)-HA3, leu2::Gal1p-Sic1(9m)/His3p-Gal1/His3p-Gal2/Gal1p-Gal4::Leu2	This study	B3427
	MATa, , SCC1-PK9::KANMX, Δscc2::NATMX4, Δlys2:scc2T1175C_HIS6_FLAG6, Δmet15::smc3(K112Q_K113Q_Q67C_R1008I_W483R)-HA3, leu2::Gal1p-Sic1(9m)/His3p-Gal1/His3p-Gal2/Gal1p-Gal4::Leu2	This study	B3363
7C and S7E	*Mat a, SCC1-PK6::TRP1, Ura3::smc3K1004C_HA3::URA3, scc2_D369C_FLAG6::KanMx, leu2::Gal1p-Sic1(9m)/His3p-Gal1/His3p-Gal2/Gal1p-Gal4::Leu2*	This study	B3814
	Mat a, SCC1-PK6::TRP1, Ura3::smc3K112Q_K113Q_K1004C_HA3::URA3, scc2_ D369C_FLAG6::KanMx, leu2::Gal1p-Sic1(9m)/His3p-Gal1/His3p-Gal2/Gal1p-Gal4::Leu2	This study	B3815
	Mat a, SCC1-PK6::TRP1, Ura3::smc3K112Q_K113Q_K1004C_R1008I-HA3::URA3, scc2_ D369C_FLAG6::KanMx, leu2::Gal1p-Sic1(9m)/His3p-Gal1/His3p-Gal2/Gal1p-Gal4::Leu2	This study	B3816
	Mat a, SCC1-PK6::TRP1, Ura3::smc3K112Q_K113Q_K1004C_W483R-HA3::URA3, scc2_ D369C_FLAG6::KanMx, leu2::Gal1p-Sic1(9m)/His3p-Gal1/His3p-Gal2/Gal1p-Gal4::Leu2	This study	B3817
	Mat a, SCC1-PK6::TRP1, Ura3::smc3K112Q_K113Q_K1004C_W483R_R1008I-HA3::URA3, scc2_D369C_FLAG6::KanMx, leu2::Gal1p-Sic1(9m)/His3p-Gal1/His3p-Gal2/Gal1p-Gal4::Leu2	This study	B3818
7F	*MAT a/alpha, Δsmc3::HIS3/SMC3, ura3/ura3::smc3(K112Q_K113Q_R1008I)::URA3, scc2(E822K, L937F):: NATMX / SCC2*	This study	B1392
7G	*Mat a, ura3::SMC3-PK6::URA3*	This study	B4002
	Mat a, ura3::smc3(K112Q_K113Q)-PK6:: URA3	This study	B4003
	Mat a, ura3::smc3(K112Q_K113Q_R1008I)-PK6:: URA3	This study	B3945
	Mat a, ura3::smc3-(K112Q_K113Q)-PK6:: URA3 ,Scc2(E822K, L937F):: NatMX	This study	B4005
	Mat a, ura3::smc3(K112Q_K113Q_R1008I)-PK6:: URA3, Scc2(E822K, L937F):: NatMX	This study	B4006
S7B	*MATa, SCC1-PK9::KANMX, SCC2_6xHis_FLAG6::KANMX, pBH585 (smc3-Q67TAG-HA3), pBH61 (tRNATyr/Bpa synthetase)*	This study	B1505

	MATa, SCC1-PK9::KANMX,, SCC2_6xHis_FLAG6::KANMX, pBH623 (smc3QQ -Q67TAG-HA3), pBH61 (tRNATyr/Bpa synthetase)	This study	B2762
	MATa, SCC1-PK9::KANMX, SCC2_6xHis_FLAG6::KANMX pBH995(smc3QQ -Q67TAG-R1008I-HA3), pBH61 (tRNATyr/Bpa synthetase)	This study	B2757
	MATa, SCC1-PK9::KANMX, SCC2_6xHis_FLAG6::KANMX, pBH998 (smc3-Q67TAG-W483R-HA3), pBH61 (tRNATyr/Bpa synthetase)	This study	B2760
	MATa, SCC1-PK9::KANMX, SCC2_6xHis_FLAG6::KANMX, pBH999 (smc3-Q67TAG-W483R-R1008I-HA3), pBH61 (tRNATyr/Bpa synthetase)	This study	B2761
S7C	*Mat a, SCC1-PK9::KANMX, Δmet15::smc3S72C_HA3, Δscc2::NATMX4, Δlys2::scc2E819C_HIS6_FLAG6,, leu2::Gal1p-Sic1(9m)/His3p-Gal1/His3p-Gal2/Gal1p-Gal4::Leu2*	This study	B3371
	Mat a, SCC1-PK9::KANMX, Δmet15::smc3(K112Q_K113Q_S72C)-HA3, Δscc2::NATMX4, Δlys2::scc2E819C_HIS6_FLAG6, leu2::Gal1p-Sic1(9m)/His3p-Gal1/His3p-Gal2/Gal1p-Gal4::Leu2	This study	B3417
	Mat a, SCC1-PK9::KANMX, Δmet15::smc3(K112Q_K113Q_S72C_R1008I)-HA3, Δscc2::NATMX4, Δlys2::scc2E819C_HIS6_FLAG6,, leu2::Gal1p-Sic1(9m)/His3p-Gal1/His3p-Gal2/Gal1p-Gal4::Leu2	This study	B3593
	Mat a, SCC1-PK9::KANMX, Δmet15::smc3(K112Q_K113Q_S72C_W483R)-HA3, Δscc2::NATMX4, Δlys2::scc2E819C_HIS6_FLAG6,, leu2::Gal1p-Sic1(9m)/His3p-Gal1/His3p-Gal2/Gal1p-Gal4::Leu2	This study	B3589
	Mat a, SCC1-PK9::KANMX, Δmet15::smc3(K112Q_K113Q_S72C_W483R_R1008I)-HA3, Δscc2::NATMX4, Δlys2::scc2E819C_HIS6_FLAG6,, leu2::Gal1p-Sic1(9m)/His3p-Gal1/His3p-Gal2/Gal1p-Gal4::Leu2	This study	B3591
S7G	*MATa/alpha, SMC3/Δsmc3::HIS3, ura3/ura3::Smc3(K112Q_K113Q_R1008I)::URA3, SCC2/Scc2(E822K):: NATMX*	This study	B3206
	MATa/alpha, SMC3/Δsmc3::HIS3 ura3/ura3::Smc3(K112Q_K113Q_R1008I)::URA3 , SCC2/scc2(L937F):: NATMX	This study	B3207

,

Supplementary Table 4 Positions of yeast CDE III

chrI	151569	151571
chrII	238310	238312
chrIII	114488	114490
chrIV	449808	449810
chrV	152091	152093
chrVI	148614	148616
chrVII	496931	496933
chrVIII	105597	105599
chrIX	355732	355734
chrX	436318	436320
chrXI	440140	440142
chrXII	150839	150841
chrXIII	268136	268138
chrXIV	628862	628864
chrXV	326595	326597
chrXVI	556060	556062

**Supplementary Table 5** antibodies and reagents		
**REAGENT or RESOURCE**	**SOURCE**	**IDENTIFIER**
**Antibodies**		
Anti-V5 (Mouse) (Dilution 1:3000)	BioRad	Cat# MCA1360
Anti-HA 3F10 (Rat) (Dilution 1:3000)	Sigma	Cat# 11867423001
Anti-PGK1 22C5D8 (Mouse) (Dilution 1:5000)	ThermoFisher	Cat# 459250
Anti-MYC 4A6 (Mouse) (Dilution 1:3000)	Sigma	Cat# 05-72405-724
Anti-FLAG M2 (Mouse) (Dilution 1:3000)	Sigma	Cat# F3165
Goat anti-Mouse IgG (H/L):HRP (Dilution 1:3000)	BioRad	Cat# STAR207
**Chemicals, Peptides, and Recombinant Proteins**		
BPA	Bachem	Cat# 4017646.0005
BMOE	ThermoFisher	Cat# 10250014
Acid-washed glass beads	Sigma	Cat# G8722
Complete EDTA free protease inhibitor cocktail	Roche	Cat# 4693132001
Indole-3-acetic acid (IAA/Auxin)	Sigma	Cat# I3750-5G-A
Immobilon Western ECL	Sigma	Cat# WBLKS0500
PMSF	Sigma	Cat# 329-98-6
Proteinase K	Roche	Cat# 03115836001
RNase A	Roche	Cat# 10109169001
-factor peptide	CRUK	N/A
NuPAGE 3-8% Tris-Acetate Protein Gels, 1.5 mm, 10-well	ThermoFisher	Cat# EA0378BOX
NuPAGE 4-12% Bis-Tris Protein Gels, 1.0 mm, 10-well	ThermoFisher	Cat# NP0321BOX
Protein G dynabeads	ThermoFisher	Cat# 10003D
StrepTrap HP	Fisher Scientific	Cat# 11540654
Desthiobiotin	Fisher Scientific	Cat# 12753064
Superose 6 Increase 10/300 GL	VWR	Cat# 29-0915-96
HiLoad 16/60 Superdex 200	GE Healthcare	Cat# GE28-9893-35
Cellfectin II Reagent	Invitrogen	Cat# 10362100
Antibiotics Antimycotic Solution	Sigma Aldrich	Cat# A5955
Fetal Bovine Serum	Sigma Aldrich	Cat# 12303C
SF-900 III SFM	Life technologies	Cat# 12658027
Ni Sepharose 6 Fast Flow	GE Healthcare	Cat# 17-5318-02
Glutathione Sepharose 4B	GE Healthcare	Cat#17075605
AxyPrep Mag PCR Clean-up kit	Appleton Woods Ltd	Cat# AX402
ChIP Clean and Concentrator kit	Zymo Research	Cat# D5205
E-Gel SizeSelect II Agarose Gels, 2%	ThermoFisher	Cat# G661012
EnzChek phosphate assay kit	Invitrogen	Cat# E6646
Library Quantification Kit Ion Torrent Platforms	KAPA Biosystems	Cat# KR0407
NEBNext Fast DNA Library Prep Set for Ion Torrent kit	New England Biolabs	Cat# E6270L
**Experimental Models: Cell Lines**		
Sf9 cells in Sf-900 II SFM	ThermoFisher	Cat# 11496015
