# Supplementary material for: Conformational dynamics of cohesin/Scc2 loading complex are regulated by Smc3 acetylation and ATP binding
Source: Nat Commun. 2023 Sep 22;14:5929. doi: 10.1038/s41467-023-41596-w (PMC10516938; doi:10.1038/s41467-023-41596-w)
Supplement: Supplementary file 3 — Reporting Summary [file 41467_2023_41596_MOESM3_ESM.pdf]

nature portfolio

Corresponding author(s): Bin HU

Last updated by author(s): Aug 18, 2023

Reporting Summary

Nature Portfolio wishes to improve the reproducibility of the work that we publish. This form provides structure for consistency and transparency in reporting. For further information on Nature Portfolio policies, see our Editorial Policies and the Editorial Policy Checklist.

Statistics

For all statistical analyses, confirm that the following items are present in the figure legend, table legend, main text, or Methods section.

n/a	Confirmed	
☐	☒	The exact sample size (*n*) for each experimental group/condition, given as a discrete number and unit of measurement
☐	☒	A statement on whether measurements were taken from distinct samples or whether the same sample was measured repeatedly
☒	☐	The statistical test(s) used AND whether they are one- or two-sided<br>*Only common tests should be described solely by name; describe more complex techniques in the Methods section.*
☒	☐	A description of all covariates tested
☒	☐	A description of any assumptions or corrections, such as tests of normality and adjustment for multiple comparisons
☒	☐	A full description of the statistical parameters including central tendency (e.g. means) or other basic estimates (e.g. regression coefficient) AND variation (e.g. standard deviation) or associated estimates of uncertainty (e.g. confidence intervals)
☒	☐	For null hypothesis testing, the test statistic (e.g. *F*, *t*, *r*) with confidence intervals, effect sizes, degrees of freedom and *P* value noted<br>*Give P values as exact values whenever suitable.*
☒	☐	For Bayesian analysis, information on the choice of priors and Markov chain Monte Carlo settings
☒	☐	For hierarchical and complex designs, identification of the appropriate level for tests and full reporting of outcomes
☒	☐	Estimates of effect sizes (e.g. Cohen's *d*, Pearson's *r*), indicating how they were calculated

Our web collection on statistics for biologists contains articles on many of the points above.

Software and code

Policy information about availability of computer code

Data collection	All the western blot data were collected using G:Box Chemi-XX9 (GENESYS) custom software; Sequencing data was produced by the Ion Torrent Proton (Life Technologies),
Data analysis	Data analysis is described in the Methods section. The density of western blot was measured using GeneTools (version 4.3.8.0, GENESYS). The analysis of ChIP-seq was performed in the Galaxy platform (the University of Aberdeen) and ChIP-seq profiles were visualised using the IGB browser (version 9.1.10). The protein structure models were developed using the program Chimera 1.14. Statistical analysis was done in Minitab Statistical Software 21.1.1 (64-bit).

For manuscripts utilizing custom algorithms or software that are central to the research but not yet described in published literature, software must be made available to editors and reviewers. We strongly encourage code deposition in a community repository (e.g. GitHub). See the Nature Portfolio guidelines for submitting code & software for further information.

Data

Policy information about availability of data

All manuscripts must include a data availability statement. This statement should provide the following information, where applicable:

- Accession codes, unique identifiers, or web links for publicly available datasets
- A description of any restrictions on data availability
- For clinical datasets or third party data, please ensure that the statement adheres to our policy

The calibrated ChIP-seq data (raw and analysed) were deposited to GEO under accession code GSE217833 (https://www.ncbi.nlm.nih.gov/geo/query/acc.cgi?acc=GSE217833). All the other original/analysed data including the modelled structures were deposited to figshare: https://doi.org/10.6084/m9.figshare.22664902.v2. The Cryo-EM structure of Scc2/J-cohesin was obtained from EMD (EMD-12880 [https://pdbj.org/emnavi/quick.php?id=emdb-12880]). The Cryo-EM structure of Scc2/E-cohesin/DNA was obtained from PDB (PDB 6ZZ6 [https://www.rcsb.org/structure/6zz6]). The crystal structure of Smc3-Scc1 was obtained from PDB (PDB 4UX3 [https://www.rcsb.org/structure/4ux3]). The crystal structure of Smc1-Scc1 was obtained from PDB (PDB 1W1W [https://www.rcsb.org/structure/1W1W]). The source data underlying main and supplementary figures are provided as a Source Data file. Additional details on datasets and protocols used in this study will be made available by the corresponding author upon reasonable request.

Research involving human participants, their data, or biological material

Policy information about studies with human participants or human data. See also policy information about sex, gender (identity/presentation), and sexual orientation and race, ethnicity and racism.

Reporting on sex and gender	not applicable
Reporting on race, ethnicity, or other socially relevant groupings	not applicable
Population characteristics	not applicable
Recruitment	not applicable
Ethics oversight	not applicable

Note that full information on the approval of the study protocol must also be provided in the manuscript.

Field-specific reporting

Please select the one below that is the best fit for your research. If you are not sure, read the appropriate sections before making your selection.

☒ Life sciences ☐ Behavioural & social sciences ☐ Ecological, evolutionary & environmental sciences

For a reference copy of the document with all sections, see nature.com/documents/nr-reporting-summary-flat.pdf

Life sciences study design

All studies must disclose on these points even when the disclosure is negative.

Sample size	In general, no calculations were done to determine sample size. Sample size was determined based on standards for experimental molecular biology studies. Typical, all western blot quantification were derived from at least three independent repeats.
Data exclusions	No data exclusion
Replication	All the experiments were repeated at least twice and the results of repeats were consistent
Randomization	No randomization was used or applicable in this research since each yeast strain with a specific genotype used in this study was derived from a single colony. Therefore, the genetic background of cells were uniform. Also, the experimental conditions such as cell culture were strictly controlled, ensuring the same physiological status of cells among different strains. The most important is that the experiments designed in this study were to examine effects under different genetic backgrounds, not different treatments. Therefore, randomization was not applicable in this study.
Blinding	Blinding was not applicable in this study, as data acquisition and quantification was performed by machines

Reporting for specific materials, systems and methods

We require information from authors about some types of materials, experimental systems and methods used in many studies. Here, indicate whether each material, system or method listed is relevant to your study. If you are not sure if a list item applies to your research, read the appropriate section before selecting a response.

Materials & experimental systems

n/a	Involved in the study
☐	☒ Antibodies
☐	☒ Eukaryotic cell lines
☒	☐ Palaeontology and archaeology
☒	☐ Animals and other organisms
☒	☐ Clinical data
☒	☐ Dual use research of concern
☒	☐ Plants

Methods

n/a	Involved in the study
☐	☒ ChIP-seq
☒	☐ Flow cytometry
☒	☐ MRI-based neuroimaging

Antibodies

Antibodies used

Anti-HA (clone 3F10): Merck (11867423001)
Anti-PK (clone SVS-Pkl): BioRad (MCA1360)
Anti-FLAG (Clone M2): Merck (F3165)
Anti-Myc (Clone 4A6): Merck (05-72405-724)
Anti-PGK1: ThermoFisher (459250)
Goat anti-Mouse IgG (H/L):HRP: BioRad (STAR207)

Validation

rat monoclonal anti-HA antibody (Merck, 11867423001, clone 3F10): one of the most commonly used antibodies against HA epitope tag in yeast, e.g., in Petela NJ et al., (2018), Scc2 is a potent activator of cohesin's ATPase that promotes loading by binding Scc1 without Pds5. Molecular Cell, 70: 1134-1148. The specificity of this antibody was also validated in my lab using untagged protein as a negative control, showing that this antibody specifically recognizes the tagged proteins.

mouse monoclonal anti-PK antibody (BioRad, MCA1360, clone SVS-Pkl): one of the most commonly used antibodies against PK epitope tag in yeast, e.g., in Petela NJ et al., (2018), Scc2 is a potent activator of cohesin's ATPase that promotes loading by binding Scc1 without Pds5. Molecular Cell, 70: 1134-1148. The specificity of this antibody was also validated in my lab using untagged protein as a negative control. No signal is detected in the negative sample, showing that this antibody specifically recognizes the tagged proteins.

mouse monoclonal anti-FLAG antibody (Merck, F3165, clone M2): one of the most commonly used antibodies against FLAG epitope tag in yeast, e.g., Gali VK, et al. (2018) Identification of Elg1 interaction partners and effects on post-replication chromatin re-formation. PLOS Genetics 14(11): e1007783. The specificity of this antibody was also validated in my lab using untagged protein as a negative control. No signal is detected in the negative sample, showing that this antibody specifically recognizes the tagged proteins.

mouse monoclonal anti-Myc antibody (Merck, 05-72405-724, clone 4A6): one of the commonly used antibodies against Myc epitope tag in yeast, e.g., Petela NJ et al., (2018), Scc2 is a potent activator of cohesin's ATPase that promotes loading by binding Scc1 without Pds5. Molecular Cell, 70: 1134-1148. The specificity of this antibody was also validated in my lab using untagged protein as a negative control. No signal is detected in the negative sample, showing that this antibody specifically recognizes the tagged proteins.

mouse monoclonal anti- PGK1 antibody (ThermoFisher, 459250, clone 22C5D8): one of the most commonly used antibodies against PGK1 in yeast, e.g., in Petela NJ et al., (2018), Scc2 is a potent activator of cohesin's ATPase that promotes loading by binding Scc1 without Pds5. Molecular Cell, 70: 1134-1148. The specificity of this antibody was also validated in my lab by western blot, showing a single band with a molecular weight of 45KDa, corresponding to the PGK1 protein.

Goat anti-Mouse IgG (H/L):HRP (BioRad, STAR207): one of the most commonly used secondary antibodies against mouse primary antibody, e.g., Gali VK, et al. (2018) Identification of Elg1 interaction partners and effects on post-replication chromatin re-formation. PLOS Genetics 14(11): e1007783. The specificity of this antibody was also validated in my lab by Western blot in which a primary antibody was not used. No signal is detected, showing that this antibody specifically recognizes mouse primary antibody.

Eukaryotic cell lines

Policy information about cell lines and Sex and Gender in Research

Cell line source(s)	Gibco™ Sf9 cells are purchased from ThermoFisher.
Authentication	We did not authenticate the cell line
Mycoplasma contamination	As stated by the supplier, the Master Seed Bank has been tested for contamination of bacteria, yeast, mycoplasma and virus and has been characterized by isozyme and karyotype analysis. We did not test mycoplasma contamination.
Commonly misidentified lines (See ICLAC register)	none

Plants

Seed stocks | No applicable

Novel plant genotypes | No applicable

Authentication | No applicable

ChIP-seq

Data deposition

☒ Confirm that both raw and final processed data have been deposited in a public database such as GEO.

☒ Confirm that you have deposited or provided access to graph files (e.g. BED files) for the called peaks.

Data access links<br>*May remain private before publication.*	GSE217833
Files in database submission	Sequencing data: Fig1C_K17407IP, Fig1C_K17407WCE, Fig1C_K22703IP, Fig1C_K22703WCE, Fig1C_K22705IP, Fig1C_K22705WCE, Fig1F_B910IP, Fig1F_B910WCE, Fig1F_B22697IP, Fig1F_B2269WCE, Fig1F_B2851IP, Fig1F_B2851WCE, Fig6G_B4002IP, Fig6G_B4002WCE, Fig6G_B4003IP, Fig6G_B4003WCE, Fig6G_B3945IP, Fig6G_B3945WCE, Fig6G_B4005IP, Fig6G_B4005WCE, Fig6G_B4006IP, Fig6G_B4006WCE<br>Analysis Data: Fig1C_K17407.bigwig, Fig1C_K22703IP.bigwig, Fig1C_K22705IP.bigwig, Fig1F_B910.bigwig, Fig1F_B2269IP.bigwig, Fig1F_B2851IP.bigwig, Fig6G_B4002.bigwig, Fig6G_B4003.bigwig, Fig6G_B3945.bigwig, Fig6G_B4005.bigwig, Fig6G_B4006.bigwig
Genome browser session<br>(e.g. UCSC)	These are calibrated ChIP-seq and can not be directly visualized using public Genome browser. We produced the bigwig files which can be visualized using IGB

Methodology

Replicates	all the experiments was repeated at least twice
Sequencing depth	The sequencing of the WCE samples produced 2-8 million reads and the sequencing of the IP samples produced 4-10 million reads. About 80-95% reads were mapped to the experimental or reference genome.
Antibodies	Anti-PK (clone SVS-Pkl): BioRad (MCA1360)
Peak calling parameters	For analyzing calibrated ChIP-seq of cohesin, peak calling is not applicable.
Data quality	The quality of the reads was assessed using FastQC (Galaxy tool version 1.,0.0) and the low-quality bases were removed as required using 'trim sequences' (Galaxy tool version 1.0.0). Generally, this involved removing the first 10 bases and any bases after the 200th but trimming more or fewer bases may be required to ensure the removal of kmers and that the per-base sequence content is equal across the reads. Reads shorter than 50 bp were removed using Filter FASTQ (Galaxy tool version 1.0.0, minimum size: 50, maximum size: 0, minimum quality: 0, maximum quality: 0, maximum number of bases allowed outside of quality range: 0, paired end data: false)
Software	The analysis of calibrated ChIP-seq were described in the paper.<br>The Galaxy platform (University of Aberdeen) was used to perform ChIP-seq data analysis.<br>FastQC (Galaxy tool version 1.0.0) was used to assess the quality of the reads.<br>Trim sequences (Galaxy tool version 1.0.0) was use to remove the low-quality bases.<br>Filter FASTQ (Galaxy tool version 1.0.0) was used to remove reads shorter than 50 bp.<br>Bowtie2 (Galaxy tool version 0.2) was used to align the remaining reads S. cerevisiae genome: SacCer3 or C. glabrata (CBS138, genolevures) genome.<br>BAM to BigWig (Galaxy tool version 0.1.0) was used to convert BAM file to BigWig file<br>IGB browser was used to visualise the BigWig file and the track was multiplied by the samples occupancy ratio (OR) using the graph multiply function.<br>ComputeMatrix (Galaxy tool version 1.0.0) was used to calculate the average occupancy of the 10kb region around all 16 centromeres.<br>A BED file named "yeast CDEIII" is used to define the centres of all the 16 CDEIII in this calculation (Table S3).
